# An efficient RNA interference screening strategy for gene functional analysis

**DOI:** 10.1186/1471-2164-13-491

**Published:** 2012-09-18

**Authors:** Chih-Hung Chang, Hsiang-Iu Wang, Hsiang-Chia Lu, Cheng-En Chen, Hong-Hwa Chen, Hsin-Hung Yeh, Chuan Yi Tang

**Affiliations:** 1 Department of Computer Science, National Tsing Hua University, Hsinchu, 30013, Taiwan; 2 Department of Plant Pathology and Microbiology, National Taiwan University, Taipei, 10617, Taiwan; 3 Department of Life Sciences, National Cheng Kung University, Tainan, 701, Taiwan; 4 Orchid Research Center, National Cheng Kung University, Tainan, 701, Taiwan; 5 Institute of Tropical Plant Scicenes, National Cheng Kung University, Tainan, 701, Taiwan; 6 Research Center for Plant Medicine, National Taiwan University, Taipei, 10617, Taiwan; 7 Department of Computer Science and Information Engineering, Providence University, Taichung, 43301, Taiwan

**Keywords:** RNA interference, RNAi screening, SiRNA design, Gene functional analysis, Group testing

## Abstract

**Background:**

RNA interference (RNAi) is commonly applied in genome-scale gene functional screens. However, a one-on-one RNAi analysis that targets each gene is cost-ineffective and laborious. Previous studies have indicated that siRNAs can also affect RNAs that are near-perfectly complementary, and this phenomenon has been termed an off-target effect. This phenomenon implies that it is possible to silence several genes simultaneously with a carefully designed siRNA.

**Results:**

We propose a strategy that is combined with a heuristic algorithm to design suitable siRNAs that can target multiple genes and a group testing method that would reduce the number of required RNAi experiments in a large-scale RNAi analysis. To verify the efficacy of our strategy, we used the *Orchid* expressed sequence tag data as a case study to screen the putative transcription factors that are involved in plant disease responses. According to our computation, 94 qualified siRNAs were sufficient to examine all of the predicated 229 transcription factors. In addition, among the 94 computer-designed siRNAs, an siRNA that targets both TF15 (a previously identified transcription factor that is involved in the plant disease-response pathway) and TF21 was introduced into orchids. The experimental results showed that this siRNA can simultaneously silence TF15 and TF21, and application of our strategy successfully confirmed that TF15 is involved in plant defense responses. Interestingly, our second-round analysis, which used an siRNA specific to TF21, indicated that TF21 is a previously unidentified transcription factor that is related to plant defense responses.

**Conclusions:**

Our computational results showed that it is possible to screen all genes with fewer experiments than would be required for the traditional one-on-one RNAi screening. We also verified that our strategy is capable of identifying genes that are involved in a specific phenotype.

## Background

The study of plant genomes has increased dramatically over the last decade [1,2]. Many genome sequencing projects for important crops, such as maize [3,4], wheat [5] and rice [6,7], have recently been completed. The available plant genomic sequence data will continue to increase explosively because of the recently developed next-generation sequencing techniques [8-10]. Therefore, a major challenge to all biologists is how to efficiently translate the functions of the genetic code sequences. It is especially important to rapidly screen for genes that are related to certain important phenotypes, such as salt-tolerance, disease-resistance and flower color, because these genes will help to improve crops. For example, by transferring *mannitol-1-phosphate dehydrogenase gene* from *Escherichia coli*, researchers have created many genetically modified plants with increased salt tolerance, which is beneficial to farmers and consumers [11,12].

One of the most efficient tools in high-throughput gene functional screening is the application of RNA interference (RNAi) [13]. RNAi can be induced by introducing a small double-stranded (ds) RNA into a cell [14] and has been widely employed in genome-wide scale studies of model organisms, including animals and plants [15-20]. The primary goals of these applications are usually related to the screening of a single gene or multiple genes that convey(s) a specific phenotype. In these applications, each dsRNA is designed to target one specific gene and to facilitate the observation of the resulting phenotype change. This one-on-one approach works very well for model organisms, because whole genome RNAi libraries are available for these model organisms [21-23]. However, even when one-on-one RNAi experiments are feasible in model organisms, it is still laborious and costly because more than ten thousand coding and non-coding genes are typically found in an organism (e.g., approximately 20,000-25,000 genes were identified in the human genome [24] and approximately 25,500 genes were predicted in the Arabidopsis genome [25]). Thus, matches to the gene number in RNAi experiments should at least be performed. Although using a vector carrying multiple shRNAs/multi-miRNA hairpins can be applied to silence multiple genes simultaneously (“combinatorial RNAi”) [26,27] and can reduce the required experiments for high-throughput analysis, these approaches still need to design and synthesize as many siRNAs as the number of target gene sets.

RNAi libraries are not available for non-model organisms, and the cost and scale of high-throughput RNAi screening experiments are still difficult for most laboratories. In reality, RNAi screening can focus on screening specific groups of genes through strategies such as literature surveys or transcriptome analyses [28,29]. However, these approaches usually identify hundreds to thousands of candidate genes; the subsequent large-scale RNAi analysis would be still a challenge for most laboratories by using a one-on-one approach. In fact, siRNAs do not need to perfectly complement their targets. Genes that contain a sequence that is partially similar to the designed siRNA could also be down-regulated (off-target effect) [30]. The off-target effects have been widely discussed and studied in both animals and plants [13,31,32] , and they are widely considered to be a general problem for analyses; however, off-target effects also facilitate the silencing of multiple genes simultaneously with a carefully designed siRNA. Thus, utilizing the off-target effect to carefully design siRNA could provide opportunities for us to design a minimum of siRNA(s) to target a maximum of genes, which would reduce the labors and costs for high-throughput gene functional screening and would decrease the obstacles for most laboratories in facing high-throughput screening. This approach would be especially useful for applications on non-model organisms for which RNAi libraries are not available.

Here, we utilize the off-target effects to develop a hierarchical RNAi screening strategy to reduce the cost and labor of high-throughput RNAi analyses. Our strategy utilizes a heuristic algorithm to design siRNAs that have multiple target genes and uses group testing methods [33] to minimize the RNAi analysis for identifying genes that are involved in a specific phenotype. Our strategy can be applied to whole-genome RNAi analyses to reduce the number of initial experiments that are needed for phenotype screening, and it is especially suitable for a group of candidate genes that were first identified by an experimental approach. Moreover, to our knowledge, no design of an siRNA for multiple-gene targets has been described previously. We have verified the applicability of this method by analyzing transcription factors that are known to be involved in plant defense responses in orchids, and we also identified a new transcription factor that is involved in this process.

## Methods

### *Orchid* ESTs library

To verify the applicability of our approach, we used an established *Orchid* (*Phalaenopsis equestris*) expressed sequence tags (ESTs) library in our initial test. There are 233 previously predicted transcription factor (TF) sequences [34] and 8017 mRNA sequences that are available for download from the NCBI ESTs database. We used the BLAST tool [35] to remove the redundant sequences. First, we used each transcription factor sequence to perform a BLAST search against the transcription factor data to delete the redundant sequences. If the BLAST result revealed that two sequences had a >95% match ratio and a >85% coverage ratio, we removed the shorter sequence as a redundant sequence. The match ratio is defined as the ratio of the number of identical bases in the aligned sequences to the total length of the aligned sequences, and the coverage ratio is defined as the total length of the aligned sequences relative to the length of the longer sequence among the two transcription factor sequences. After the pre-processing, there were 229 non-redundant transcription factor (TF) sequences remaining. The sequences that we used are available at OrchidBase ( http://140.116.25.218/EST/) [36].

Because the mRNA data could overlap with TF data, we continued to remove the mRNA sequences that overlapped with the TF data. We performed a BLAST search against the mRNA data for each TF sequence. If the BLAST result revealed that one mRNA sequence had an >85% coverage ratio or 90% overlapping ratio with the TF sequence, then we removed the mRNA sequence as an overlapping sequence. The overlapping ratio was defined as the total length of the aligned sequences relative to the length of the shorter sequence among these two sequences.

#### Qualified siRNA model for our strategy

Our strategy was designed to screen all of the genes in the genome or to screen candidate genes that had been narrowed down using previous data or experimental analyses. Therefore, without loss of generality, we defined two disjoint sets of genes. We first defined the set of candidate-genes, *C* = {*c*_*1*_, *c*_*2*_,…, *c*_*n*_}, which contained the candidate genes of the RNAi analysis. Second, we defined the set of excluded-genes, *E* = {*e*_*1*_, *e*_*2*_,…, *e*_*m*_}, which contained all of the genes except the candidate-genes. Because silencing these excluded-genes would have likely complicated further analysis, our RNAi design attempted to target the candidate-genes and avoid silencing the excluded-genes. Furthermore, to utilize the off-target effect to design the proper siRNAs for the analysis, we employed two user-defined parameters, *d*_*T*_ and *d*_*N*_, to determine the specificity of the designed siRNAs. First, the user can employ *d*_*T*_ to define the maximum number of sequence mismatches that will be tolerated between the designed siRNA and its target. Second, the user can adjust *d*_*N*_ to define the minimum number of sequence mismatches that will be allowed between the designed siRNA and its non-target to prevent the designed siRNA from targeting unanticipated genes. Before we can provide a formal definition for a qualified siRNA and its target gene(s), we must first introduce the definition of a qualified sequence as follows:

Given a set of *n* candidate-genes, *C* = {*c*_*1*_, *c*_*2*_,…, *c*_*n*_}, a set of *m* excluded-genes, *E* = {*e*_*1*_, *e*_*2*_,…, *e*_*m*_}, and three integers, *d*_*T*_, *d*_*N*_ and *L* (*L* >*d*_*N*_>*d*_*T*_), a sequence *r* of length *L* is determined to be a qualified sequence if and only if there exists a subset of candidate-genes *T*, *T* ⊆ *C*, such that for each gene *t*_*i*_ ∈ *T*, *HD*(*r*, *u*_*i*_) _≤_*d*_*T*_ for some length *L* substring *u*_*i*_ of *t*_*i*_ and for each gene *g*_*i*_ ⊆ {*E ∪ C−T*}, *HD*(*r*, *u′*_*i*_) _≥_*d*_*N*_ for any length *L* substring *u′*_*i*_ of *g*_*i*_. Here *HD*(,) represents the Hamming distance between two strings.

We defined a qualified siRNA *r*^′^ as the reverse complement sequence of a qualified sequence. Therefore, by definition, finding a qualified siRNA is equal to finding a qualified sequence. Moreover, because there are no more than *d*_*T*_ mismatches between each gene *t*_*i*_ ∈ *T* and the qualified siRNA *r*^′^, *T* would be the target gene set of the qualified siRNA *r*^′^. Conversely, {*E* ∪ *C−T*} is the non-target gene set of *r*^′^ because each gene *g*_*i*_ ⊆ {*E ∪ C−T*} has at least *d*_*N*_ mismatches with the qualified siRNA *r*^′^. Each qualified siRNA can silence some of the candidate-genes and avoids silencing the excluded-genes; therefore, they can be used to perform an RNAi experiment that will identify genes that are involved in a specific phenotype.

#### Enumerating subsequences and computing subsequence similarity

**Figure 1:**
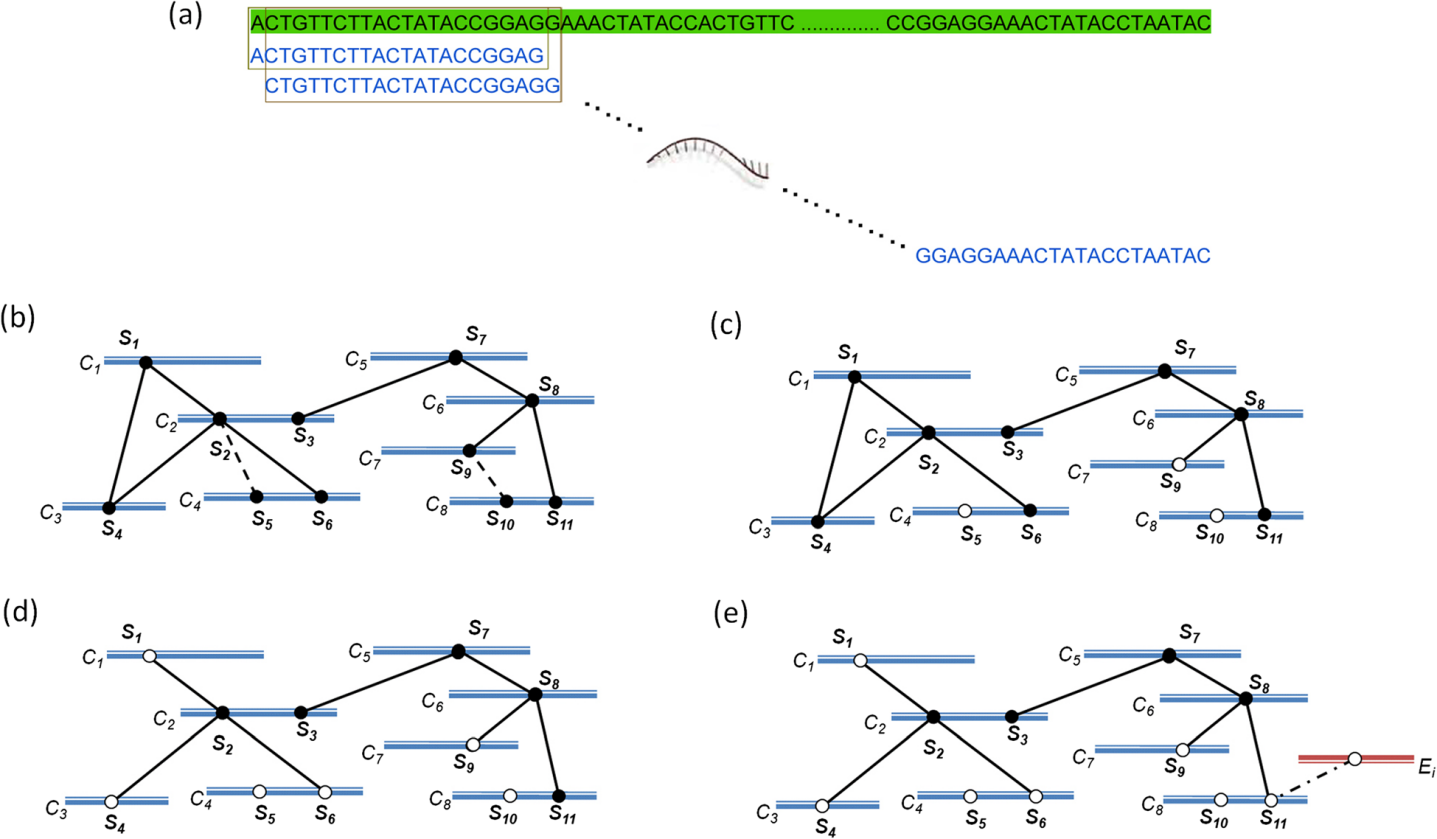
**Schematic diagram of each step of our heuristic method.** (**a**) The process of enumerating the subsequences from the candidate-genes. The green sequence represents a candidate-gene, and the blue sequences represent the subsequences that were derived from this candidate-gene by the sliding scan. (**b**) Diagram of the relationship between the subsequences. The blue lines *C*_*1*_, *C*_*2*_,…, *C*_*8*_ represent the candidate-genes, and *S*_*1*_, *S*_*2*_,…, *S*_*11*_ are the subsequences that were enumerated from these candidate-genes. A solid line between two subsequences indicates that the two subsequences are ***neighbors***, and the dotted line indicates that the two subsequences are ***far_neighbors***. Each subsequence is marked as a candidate for a qualified sequence. (**c**) Diagram of the relationship between the marked subsequences after the ***far_neighbor*** examination. The subsequences *S*_*5*_, *S*_*9*_ and *S*_*10*_ are unmarked because they all contained a ***far_neighbor***. However, *S*_*2*_ is still marked because one of its ***neighbors*** was located in *C*_*4*_, and its ***far_neighbor****S*_*5*_ was also located in *C*_*4*_. In this situation, we are not concerned about whether the siRNA that was designed based on *S*_*2*_ will recognize *S*_*5*_ because *C*_4_ is already the target gene of this siRNA. Therefore, *S*_*2*_ is still marked as a candidate for the qualified sequence. (**d**) Diagram of the relationship between the marked subsequences after the powerful subsequence examination. *S*_*1*_, *S*_*4*_ and *S*_*6*_ are unmarked because they are not powerful subsequences and are all dominated by *S*_*2*_. (**e**) Diagram of the relationship between the marked subsequences after the excluded-gene hit examination. *E*_*i*_ is one of the excluded-genes, and the dot-dashed line indicates that the Hamming distance between a marked subsequence and a substring that is located in an excluded-gene is less than *d*_*N*_; this scenario also indicates that this marked subsequence contains an excluded-gene hit. Because any subsequences that contain an excluded-gene hit will be unmarked, *S*_*11*_ is unmarked.

To obtain sequences for the qualified siRNAs, we first enumerated all of the subsequences of length *L* from the candidate-genes with a sliding scan (Figure 1a). The subsequences that contained an undetermined nucleotide “N” were discarded due to the bad sequencing quality of the sequence. Each subsequence from where the sequence was derived and its original position was recorded. Therefore, every subsequence could be differentiated by its original sequences and its position. We temporarily marked these subsequences as candidates for qualified sequences. Next, we computed the Hamming distances of the pairwise subsequences to measure their sequence similarity. If the Hamming distance between two subsequences was equal to or smaller than *d*_*T*_, these two subsequences were considered ***neighbors***. However, if the Hamming distance between the two subsequences was larger than *d*_*T*_ and smaller than *d*_*N*_, these two subsequences were considered ***far_neighbors***.

According to the sequence similarity, we obtained the neighborhood relationship of all of the similar subsequences (Figure 1b). Let *N*(*r*) denotes the set of all ***neighbors*** of the subsequence *r*. According to the off-target effect, because *r* is very similar to its ***neighbors***, when the reverse complement sequence of *r* is used as an siRNA in an RNAi experiment, it is possible that this siRNA will also target the ***neighbors*** of *r*. Therefore, the neighborhood relationship of these subsequences allowed us to determine the potential target genes of the siRNAs that were designed based on these subsequences.

### *Far_neighbor* and powerful subsequence examination

To prevent the targeting of unanticipated candidate-genes, each qualified sequence was required to contain a Hamming distance of at least *d*_*N*_ with any substrings of each gene with the exception of its own target genes. However, because *d*_*T*_ and *d*_*N*_ are defined by users, a subsequence may have ***far_neighbors***. If we designed an siRNA that was derived from a subsequence that contained ***far_neighbors***, this designed siRNA would not be guaranteed to target these ***far_neighbors***. Therefore, in this situation, our algorithm avoids selecting subsequences that contain ***far_neighbors*** to design the siRNA, and it would unmark the subsequence with ***far_neighbors*** unless the ***far_neighbors*** of a subsequence were derived from the genes from which its ***neighbors*** were derived (Figure 1c).

To reduce the computing time that was required to perform the next steps, we attempted to remove the less useful subsequences. To achieve this, we first looked for a “powerful subsequence”. Any subsequence *P* that satisfied equation **(1)**, where ∪ is the union operator, was defined as a powerful subsequence, and the powerful subsequence dominated its ***neighbors***. Because the powerful subsequence is expected to target all of its ***neighbors***' target genes, the removal of these subsequences (their target genes can be covered by the powerful subsequence) will save computational resources without causing any side effects. (Additional file 1, Figure S1 shows an example of a powerful subsequence and the subsequences that would be unmarked.)

(1) $$\cup_{v\;\in \;N\left(P\right)}N\left(v\right)\subseteq \left\{N\left(P\right)\cup P\right\}$$

To identify the powerful subsequences, we examined each subsequence to determine whether it satisfied equation (1). After identifying the powerful subsequences, we continued to unmark the subsequences that were dominated and were not powerful subsequences (Figure 1d). These unmarked subsequences were ignored in our later analysis.

#### Excluded-gene hit examination

To prevent the targeting of excluded-genes, we continued to examine whether each marked subsequence displayed a Hamming distance at least a *d*_*N*_ to every excluded-gene. For any subsequence *t*, if there existed a subsequence *u* of an excluded-gene such that *HD(t, u) < d*_*N*_, *HD(t, u)* represented the Hamming distance between *t* and *u*, then *t* is determined to contain an excluded-gene hit and this subsequence *t* would be abandoned. To perform this work, we enumerated all of the substrings of length *L* from the list of excluded-genes by utilizing a sliding scan. Next, we computed the Hamming distance between these substrings and the marked subsequences. We unmarked the subsequences that contained an excluded-gene hit (Figure 1e). The remaining marked subsequences were therefore determined to be qualified sequences, and the reverse complement sequences of these qualified sequences were qualified siRNAs.

#### Redundancy reduction

All of the qualified siRNAs are expected to silence the candidate-gene(s) without silencing the excluded-gene; however, several siRNAs could target the same genes. Because these qualified siRNAs are redundant to each other, only the effective siRNA will be selected. To properly evaluate the effective siRNA, we use existing siRNA designing rules to evaluate the effective siRNA. In plants, design rules for only artificial microRNAs, amiRNAs, (microRNAs are another class of small regulatory RNA [37]) have been proposed [38]. The proposed amiRNA targeting rules have been shown to share several features with the proposed siRNA designing rules in animal systems [39,40]. We used the siRNA designing rules proposed by Reynolds et al. [40] for selecting siRNA in our analysis. Three siRNA designing rules proposed by Reynolds [40], Hsieh [41] and Takasaki [42] are currently included in our program, allowing users to select their preferred design rules.

If more than one qualified siRNA passed the evaluation, then we continue to calculate their Hamming distance sum. The Hamming distance sum, *HD-sum* for short, is the sum of all of the Hamming distances between a qualified sequence and all of its ***neighbors***. A smaller *HD-sum* indicates that a qualified sequence is more similar to its ***neighbors***, and the qualified siRNA that is derived from it may have a higher gene silencing efficiency. Therefore, when two qualified sequences had the same target genes, we retained the qualified sequence that displayed the smaller *HD-sum*. If two qualified sequences both displayed the same *HD-sum*, we discarded the subsequences that displayed the maximum Hamming distance to one of its ***neighbors***. After we eliminated the redundancies, we obtained the non-redundant qualified sequences.

#### Construction of a pB7GWIWG2-derived clone

The primer pairs PhaTF15-hpRNA F/PhaTF15-hpRNA R, PhaTF21-hpRNA F/PhaTF21-hpRNA R, PhaTF60 hpRNA F/PhaTF60 hpRNA R and PhaRNA hpRNA F/PhaRNA hpRNA R were used to anneal to double-stranded (dsDNA) (Table 1). Two microliters of each primer (15 μg/μl) was mixed with its complementary primer at 72°C for 10 min and the solution was transferred to 25°C for an additional 10 min. The dsDNA was then cloned into the pCR8®/GW/TOPO® Gateway entry vector (Invitrogen) by following the manufacturer's recommendations. The pCR8®/GW/TOPO dsDNA construct was sequenced to confirm the sequence of the cloned fragments. The LR Clonase II enzyme (Invitrogen) was used to transfer the cloned fragments into pB7GWIWG2 [43] to generate pB7GWIWG2-PhaTF15-hpRNA. 

**Table 1:** Primers used in this study

**Primer**	**Nucleotide sequence**
TF106 F	5^′^-CCAAAACGGCCGAGGACCCC-3^′^
TF106 R	5^′^-CGCCACACACCTGACGGTCC-3^′^
TF157 F	5^′^- TCATGAACTCGTCGCCTTCCGA-3^′^
TF157 R	5^′^-GCTCGCCCTTCATACGTGGCA-3^′^
TF187 F	5^′^-TGCAAGTTCCAGCAATGCTCCT-3^′^
TF187 R	5^′^-AGAACAACAGGGATGGTGTGCATC-3^′^
PhaTF15-hpRNA F	5^′^-CCTGCGATTTCGACTATAAGT-3^′^
PhaTF15-hpRNA R	5^′^-ACTTATAGTCGAAATCGCAGG-3^′^
PhaTF21-hpRNA F	5^′^-TTACGGAGACCATCTTAAAAC-3^′^
PhaTF21-hpRNA R	5^′^-GTTTTAAGATGGTCTCCGTAA-3^′^
PhaTF60 hpRNA F	5^′^-CAACTTATGCCACTATGCAAT-3^′^
PhaTF60 hpRNA R	5^′^-ATTGCATAGTGGCATAAGTTG-3^′^
PhaRNA hpRNA F	5^′^-TGGTTCCGGTGGTGGGGGCTA-3^′^
PhaRNA hpRNA R	5^′^-TAGCCCCCTCCTCCGGAACCA-3^′^
CymMV CPF	5^′^-GAAATAATCATGGGAGAGCC-3^′^
CymMV CPR	5^′^-AGTTTGGCGTTATT CAGTAGG-3^′^
PR1 F	5^′^-AGGACCCTGGCGTCTAAAG-3^′^
PR1 R	5^′^-TATTACAAATCAAACCGCTAAAG-3^′^
NPR1 F	5^′^-CGCATTTGTGTCGTAGTTTAT-3^′^
NPR1 R	5^′^-GGCCTTGCTCCTTTAGTTA-3^′^
TF15 F	5^′^-TGAATTAGCACAAGATCCGAT-3^′^
TF15 R	5^′^-AGCAATGGAAACAGACCACCAAT-3^′^
TF21 F	5^′^-AGGGACGGAGATGCCAAGAAGGC-3^′^
TF21 R	5^′^ -CAACCATCCAGCTAATCTATC-3^′^
TF60 F	5^′^-ATGATTTCAAAATTTTATCAGTT-3^′^
TF60 R	5^′^-CAATGGAATACTATTCTGACCA-3^′^
Pha RNA F	5^′^-AAGCCGTCAAGTACAAGGGC-3^′^
Pha RNA R	5^′^-GCTAATCAGTTGCAAGAATTA-3^′^
Ubiqutin10 F	5^′^-CCGGATCAGCAAAGGTTGA-3^′^
Ubiqutin10 R	5^′^-TCAGGCGGAGGACAAGATG-3^′^

#### Transient gene silencing assay

pCambia-CymMV*-*Gateway, pB7GWIWG2 and their derivatives were transformed into *Agrobacterium tumefaciens* LBA4404 by electroporation. The *A. tumefaciens* strains were cultured in 2 ml of YEB of medium containing 100 mg/l kanamycin and 100 μM acetosyringone overnight at 28°C . One milliliter of the bacterial culture was then transferred into 10 ml of YEB medium containing 100 mg/l kanamycin and 100 μM acetosyringone and was further incubated at 28°C until the OD reached approximately 1.0–1.2. The *A. tumefaciens* cultures were then centrifuged at 3000 ×*g* for 10 min; the cells were resuspended in 1 ml of infiltration medium (10 mM MES, 10 mM MgCl_2_, 100 mM acetosyringone) and incubated at room temperature for 3 h. Each *Phalaenopsis* plant was then infiltrated with 100 μl of the *A. tumefaciens* suspension. The plant RNA was extracted and analyzed 4 days after the agroinfiltration.

#### RNA isolation and semiquantitative RT-PCR analysis

To conduct the semiquantitative RT-PCR experiment, the total RNA was extracted from the orchid plant as previously described [44]. One microgram of RNA was treated with RNase-free DNase (Ambion, 1 unit) for 0.5 h to eliminate any genomic DNA contamination. Next, 0.5 μg of DNA-free RNA for each sample was used for the synthesis of the first strand of cDNA using Moloney murine leukemia virus (MMLV) reverse transcriptase following the manufacturer's instructions (Invitrogen). The PCR amplification was performed using gene-specific oligonucleotide primers according to a previously described method [45]. The PCR products were separated by electrophoresis and visualized with ethidium bromide staining. Ubiquitin 10 was used as an internal control. The primer pairs used in the semiquantitative RT-PCR analysis are listed in Table 1.

## Results

### A case study using the orchid ESTs library

To verify the applicability of our strategy, we used an established *Orchid* (*Phalaenopsis equestris*) expressed sequence tags (ESTs) library in our initial test. The ESTs library contained 229 predicted transcription factor sequences and 7721 mRNA sequences (see the subsection “***Orchid*EST library**” in the Methods section).

**Figure 2:**
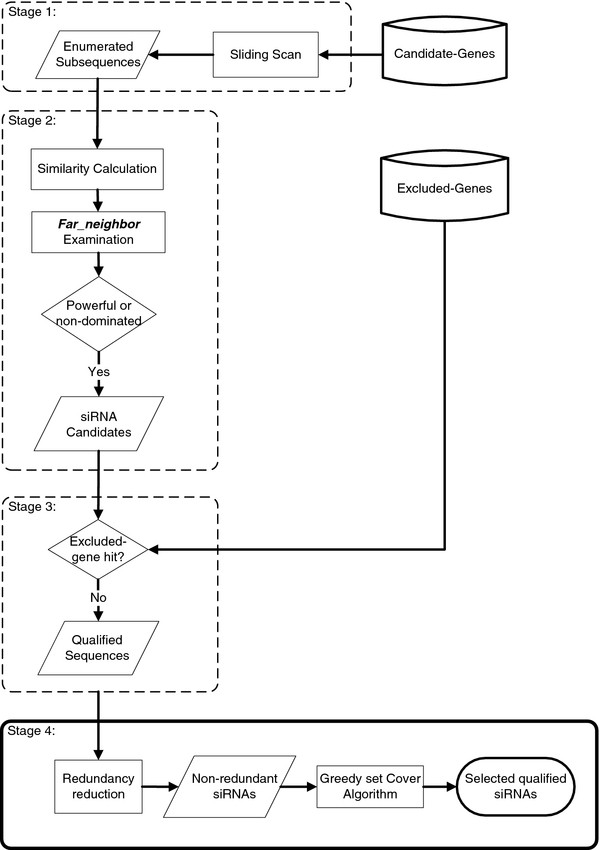
**Flowchart of the design of the qualified siRNAs that were used in the first-round analysis of our strategy.** The first three stages demonstrate the flow of the heuristic algorithm that we proposed. The qualified sequences can be derived by this heuristic algorithm, and the complement sequences of these qualified sequences are the qualified siRNAs. Based on the availability of the qualified siRNAs, we can then select the siRNAs for the first-round RNAi analysis from the qualified siRNAs in stage 4.

To use our strategy to screen for transcription factors that are involved in plant defense responses, we defined the predicated transcription factors as candidate-genes and the remaining mRNAs as excluded-genes. A previously described transcription factor, TF15, was used as a positive control [46]. Before we applied our strategy to screen for transcription factors, we first needed to design qualified siRNAs to silence the candidate-genes (see the subsection “**Qualified siRNAs for our strategy**” in the Methods section). Obtaining qualified siRNAs that target the maximum number of genes was desirable to minimize the number of experiments that were performed for the RNAi screening. However, this problem is NP-hard [we used the *distinguishing substring selection* problem (DSSP) [47,48] to prove that finding a qualified siRNA that has the maximum number of target genes is NP-complete, and the proof is presented in Additional file 2. Moreover, to analyze every candidate-gene by RNAi screening, we needed to obtain sufficient numbers of qualified siRNAs to silence all of the candidate-genes. Therefore, we needed at least *k* qualified siRNAs such that ∪_1≤*i*≤*k*_*Ti* = *C*, where *T*_*i*_ is the target gene set of siRNA *r*^′^_*i*_, and *C* is the set of candidate-genes. To obtain a sufficient number of qualified siRNAs and their corresponding target genes within a reasonable time, we provided a heuristic algorithm. This heuristic algorithm was incorporated into the workflow that was used to design the siRNAs that were needed in the first-round analysis of our strategy. The flowchart is depicted in Figure 2. 

Because previous experiments indicated that 21 nucleotides were sufficient to trigger RNAi in plants, we set the length (*L*) of the qualified siRNA to 21 [49]. Previous reports also indicated that siRNAs can target genes with four mismatches [13,50]. Therefore, we first tolerated the presence of four mismatches (*d*_*T*_) between an siRNA and its target gene. To avoid the target of unanticipated genes by the siRNA, each siRNA was designed to contain at least five mismatches (*d*_*N*_), with every gene excluding its own target genes. Therefore, in this case study, we selected the parameters *d*_*T*_ = 4, *d*_*N*_ = 5 and *L* = 21 for our heuristic algorithm to design the qualified siRNAs.

In the first stage of our heuristic algorithm, we enumerated 191,087 subsequences of length 21 from these 229 TF sequences and then computed the pairwise Hamming distances between these subsequences (see the subsection “**Enumerating subsequences and computing subsequence similarity**” in the Methods section). According to the similarity relationship of these subsequences, we obtained the information regarding the potential target genes of the siRNAs that were designed based on these subsequences. Next, to ensure that the designed siRNAs contained at least a *d*_*N*_ distance to their non-target genes and to remove the less powerful subsequences, we performed a ***far_neighbor*** and powerful subsequence examination of these subsequences, and we unmarked the disqualified subsequences (see the subsection “***Far_neighbor*and dominating subsequence examination**” in the Methods section). After this examination, 188,889 subsequences were still marked. To prevent the designed siRNAs from targeting the excluded-genes, we continued to perform the excluded-gene hit examination of the remaining marked subsequences in the third stage (see the subsection “**Excluded-gene hit examination**” in the Methods section). In total, 106,188 subsequences passed this examination, and these subsequences were the qualified sequences.

In stage 4, the subsequences were selected from the qualified sequences, and they were used as templates to design the qualified siRNAs. However, some of the qualified siRNAs that were derived from these qualified sequences may have targeted the same genes, which would have resulted in redundancy; therefore, we incorporated some pre-proposed siRNA design rules [40-42] in the redundancy reduction stage to select the best sequences for this use (see the subsection “**Redundancy reduction**” in the Methods section). After the reduction was performed, 2,147 non-redundant qualified siRNAs remained. To screen every candidate-gene at least once, we needed at least *k* qualified siRNAs, *r*_*1*_ … *r*_*k*_, such that ∪_1≤*i*≤*k*_*Ti* = *S*, where *T*_*i*_ is the target gene set of siRNA *r*_*i*_. Moreover, to minimize the cost, we also wanted to minimize the number of first-round RNAi experiments. The problem of satisfying ∪_1≤*i*≤*k*_*T*_*i*_ = *S* with a minimum of *k* qualified siRNAs is exactly the set cover problem that has been proven to be an NP-hard problem [51]. Therefore, we adopted the greedy algorithm for the set cover problem to select the siRNAs [52,53]. Furthermore, to increase the examination frequency of each candidate-gene, we slightly modified this greedy algorithm (see Additional file 3 for more detail). A total of 94 siRNAs were selected from the 2,147 non-redundant qualified siRNAs by our modified greedy algorithm (the list of the selected siRNAs is reported in Additional file 4: Table S1). According to our computational result, these 94 selected siRNAs are sufficient to examine all 229 candidate-genes in the first-round of the RNAi experiments (Table 2). Any researchers who need our program can email to us or visit the website http://algorithm.cs.nthu.edu.tw/RNAi_screening.php. 

**Table 2:** The detailed results of our testing on the Phalaenopsis ESTs data with different parameters

**Parameters**	***d***_***T***_ **= 3,*d***_***N***_ **= 5,*L*** **= 21**	***d***_***T***_ **= 4,*d***_***N***_ **= 5,*L*** **= 21**	***d***_***T***_ **= 4,*d***_***N***_ **= 6,*L*** **= 21**
**Number of enumerated subsequences**	191,087	191,087	191,087
**Number of subsequences (after*Far_neighbor*and powerful subsequence examination)**	173,106	188,889	117,203
**Number of qualified siRNAs**	100,890	106,188	6,443
**Number of non-redundant qualified siRNAs**	487	2,147	352
**Average number of target genes of a qualified siRNA**	1.0583	1.1163	1.0852
**Number of qualified siRNAs (number of target gene > 1)**	5,565	10,863	491
**Average number of target genes of qualified siRNAs (number of target gene > 1)**	2.0577	2.1370	2.1181
**Number of selected siRNAs in first round analysis**	**142**	**94**	**162**
**Number of candidate-genes examined in first round analysis**	229	229	227
**Average examination frequency of each candidate-gene**	1.153	1.170	1.110

From these result we can see that the number of qualified siRNA has important influence on the number of used siRNAs in first round analysis.

### Hierarchical RNAi analysis strategy

**Figure 3:**
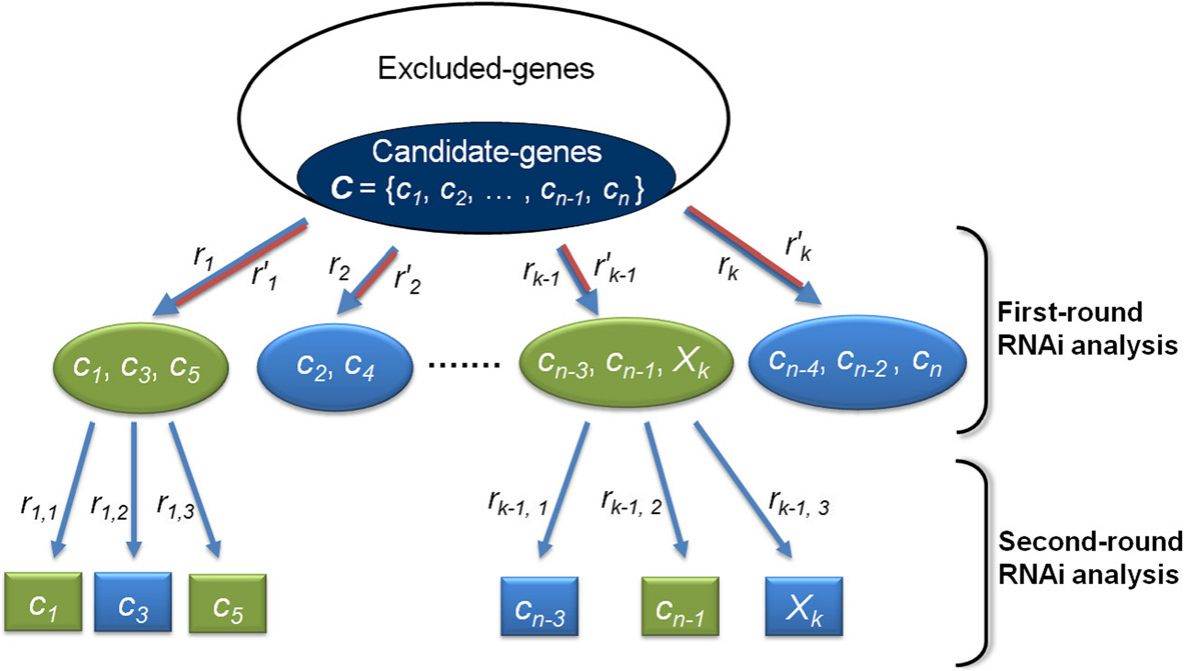
**Schematic diagram of the hierarchical RNAi analysis for identifying genes that are involved in a specific phenotype. ***R* = {*r*_*1*_, *r*_*2*_, …, *r*_*k*_} is the set of qualified siRNAs that were selected by our modified algorithm. The candidate-genes in each ellipse are the target genes of each available siRNA *r*_*i*_. In the first-round RNAi analysis, we can quickly determine which candidate-genes could be related to the target phenotype based on the RNAi experiment results. The green ellipse represents the target phenotype that was affected in the RNAi experiment with *r*_*i*_, whereas the blue ellipse indicates that the target phenotype was not affected. In the second-round RNAi analysis, we further examined the candidate-genes in the green ellipses to precisely identify the related genes. However, the complementary strand of the selected siRNA, *r*^′^_*i*_, can also be loaded into the RISC, and it could facilitate RNAi. A gene that contains a subsequence that contains no more than *d*_*T *_mismatches to *r*^′^_*i*_ would be regarded as the possible target gene of *r*^′^_*i*_, which is defined as *X*_*i*_. To precisely identify the associated gene from the genes that are affected by *r*_*i*_ and *r*^′^_*i*_, we would silence those genes one-on-one to identify which genes were definitively associated with the target phenotype.

Once we had selected the 94 siRNAs (See Additional file 4: Table S1 for more detail), we were able to perform our strategy, hierarchical RNAi analysis, which contained two rounds of RNAi analysis (Figure 3 shows the schematic diagram of the hierarchical examination process). In the first round of RNAi analysis, we observed phenotypes and correlated those phenotypes to at least one of the genes that were affected in the experiment. Thus, we can rapidly exclude the candidate-genes that did not affect the target phenotype from the suspect list, and we can also determine which candidate-genes could be related to the target phenotype. To identify the associated gene more precisely, a second round of analysis was needed. However, in an RNAi experiment, the selected siRNA and its reverse complement sequence (the selected siRNA sense strand) are employed to form a dsRNA that triggers RNAi. Previous studies have demonstrated that both of the dsRNA strands can be loaded into the RNA-induced silencing complex (RISC) and that both strands can facilitate RNAi [54-56]. Therefore, in addition to the target genes of the selected siRNA, we also needed to examine the possible target(s) of the complementary strand of the selected siRNA. The potential target genes contained a subsequence that had at most *d*_*T*_ mismatches (in this case, *d*_*T*_ = 4) with the selected siRNA sense strand. To precisely identify the associated gene from these affected genes, we designed dsRNAs that would specifically target all of the possible target genes in the second-round analysis (Figure 3). According to our statistical results from each sense strand of the qualified siRNA, the average number of potential targets of the sense strand was 0.6511; thus, only a limited number of genes could have been affected by the siRNA sense strands in each RNAi experiment in the first-round analysis. 

### Experimental verification

**Figure 4:**
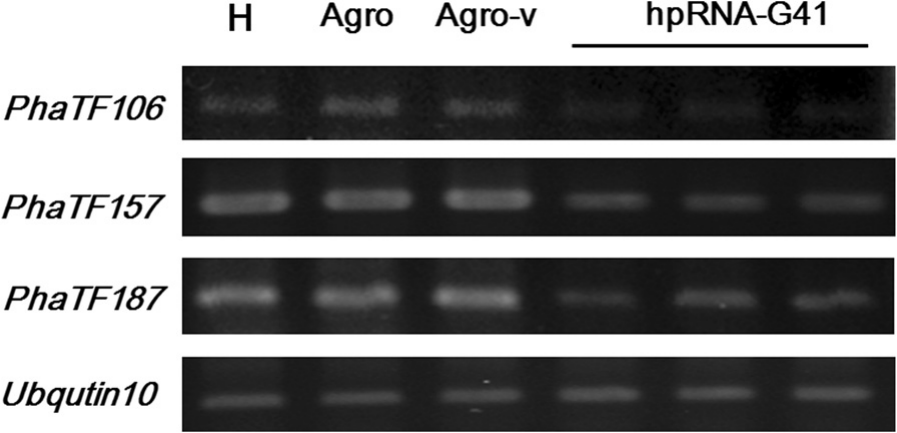
**Using siRNA-G41 to simultaneously silence three target genes.** The effects of silencing three putative transcription factors in plants that were transfected with a hairpin RNA-G41 are shown. The RNA levels of *Pha106*, *Pha157* and *PhaTF187* were analyzed in healthy (H) plants, in plants that were infiltrated with *Agrobacterium* (agro), in plants that were infiltrated with *Agrobacterium* that carried an empty pB7GWIW2 vector (agro-v) or in plants that were infiltrated with *Agrobacterium* that carried pB7GWIW2 to deliver hairpin RNAs designed from siRNA-G41 (hpRNA-G41). *Phalaenopsis Ubiquitin* 10 was used as an internal control.

To verify if siRNA can silence multiple genes with four mismatches, we first randomly selected an siRNA, siRNA-G41. The predicted targets of siRNA-G41 are TF106, TF157, and TF187. The number of mismatches between siRNA-G41 and its targets, TF106, TF157, and TF187, are 4, 0 and 2, respectively. The experimental results showed that siRNA-G41 successfully silenced *PhaTF106*, *PhaTF157* and *PhaTF187* (Figure 4), demonstrating that it is feasible to simultaneously silence multiple genes with a carefully designed siRNA and that siRNA can silence the target gene even with 4 mismatches to its target genes.

Among the 94 siRNAs, we observed one siRNA, siRNA-G55, which targeted two transcription factors, TF15 and TF21. TF15 has been previously reported to be involved in the critical salicylic acid (SA)-related plant defense response [46]; the depletion of TF15 in SA-treated plants resulted in the decreased expression of orchid pathogenesis-related gene 1 (*PhaPR1)*, which is a marker of the plant defense response, and the important central regulator *PhaNPR1*. To confirm that our strategy can help us to determine that TF15 is involved in plant defense responses, we selected siRNA-G55 to perform a first-round RNAi experiment that utilized *Agrobacterium*-mediated dsRNA delivery. The dsRNA that was derived from siRNA-G55 silenced both TF15 and TF21 but not the randomly selected transcription factor TF60.

**Figure 5:**
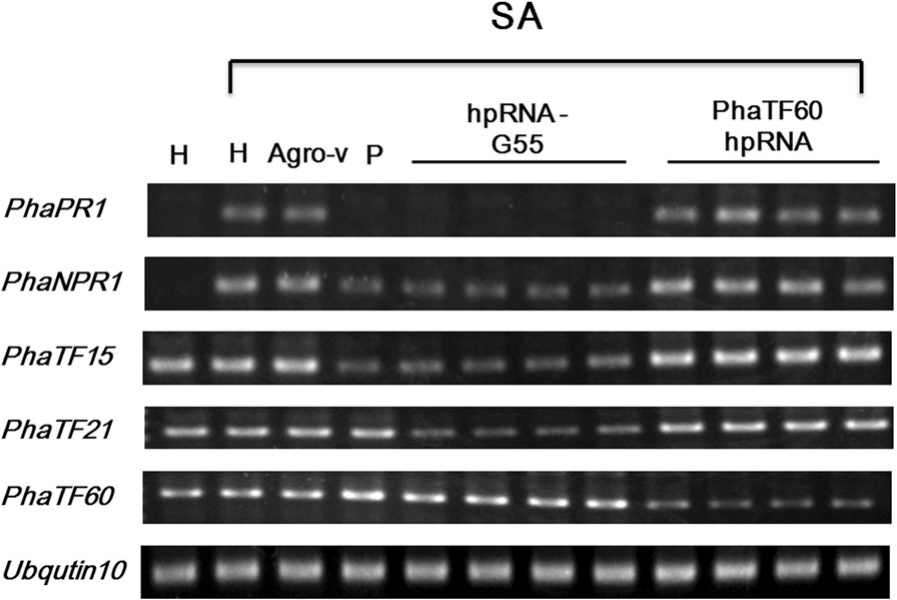
**Using siRNA-G55 in first-round analysis.** The effects of silencing the marker genes that are involved in the salicylic acid (SA)-related plant defense response pathway in plants that were transfected with a hairpin RNA-G55. The RNA levels of *PhaPR1*, *PhaNPR1*, *PhaTF15*, *PhaTF21* and *PhaTF60* were analyzed in healthy (H) plants, plants that were infiltrated with *Agrobacterium* that carried an empty pB7GWIW2 vector (agro-v), or *Agrobacterium* that carried pB7GWIW2 to deliver different hairpin RNAs that were designed from siRNA G55 (hpRNA-G55) or were specific to PhaTF60 (PhaTF60-hpRNA). The *Agrobacterium* strain that carried a partial *PhaTF15* cDNA was used as a positive control (P), and *PhaTF60* was used as a negative control. The plants that were pretreated with SA to induce the SA-related plant defense response are indicated. *Phalaenopsis Ubiquitin* 10 was used as an internal control.

The first-round experimental result showed that siRNA-G55 successfully silenced both *PhaTF15* and *PhaTF21*, and the RNA levels of *PhaPR1* and *PhaNPR1* were affected (Figure 5). This scenario verified that we could observe a change in the target phenotype when a related gene is affected in the first-round analysis. According to our strategy, we then select two siRNAs from qualified siRNAs that are specific to *PhaTF15* or *PhaTF21* for the second-round analysis. Because the complementary strand of siRNA-G55 also targeted a *Phalaenopsis* gene (*PhaRNA*, accession number CB034920 [34]), we also designed an siRNA that was specific to this RNA sequence. 

**Figure 6:**
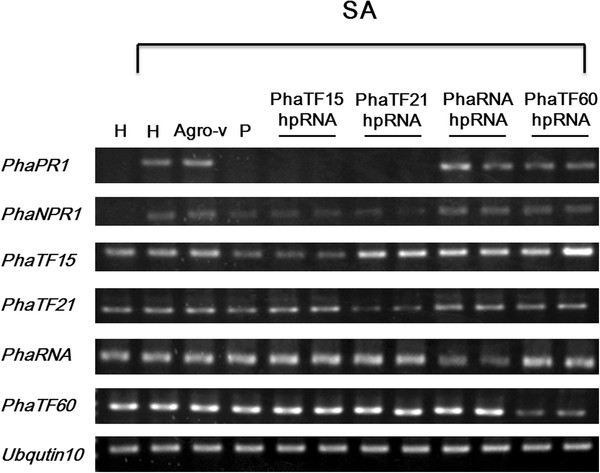
**Identifying the associated gene more precisely in the second-round analysis.** The knockdown effects of the marker genes involved in the salicylic acid (SA)-related plant defense response pathway in plants that were transfected with hairpin RNA and that are specific to *PhaTF15* and *PhaTF21*. The RNA levels of *PhaPR1*, *PhaNPR1*, *PhaTF15* and *PhaTF60* were analyzed in healthy (H) plants, plants that were infiltrated with *Agrobacterium* that carried an empty pB7GWIW2 vector (agro-v), or *Agrobacterium* that carried pB7GWIW2 to deliver different hairpin RNAs that were specific to *PhaTF15* (PhaTF15 hpRNA), *PhaTF21* (PhaTF21 hpRNA), *PhaRNA* (PhaRNA), or *PhaTF60* (PhaTF60 hpRNA). The *Agrobacterium* strain that carried the partial *PhaTF15* cDNA was used as a positive control (P), and *PhaTF60* was used as a negative control. The plants that were pretreated with salicylic acid (SA) to induce the SA-related plant defense response are indicated. *Phalaenopsis Ubiquitin* 10 was used as an internal control.

The second-round experimental data indicated that both *PhaPR1* and *PhaNPR1* were silenced in the *PhaTF15-* and *PhaTF21-*silenced plants, but not in the *PhaRNA-*silenced plant (Figure 6). These data suggested that *PhaTF15* and *PhaTF21* are involved in the SA-related plant defense response pathway but that *PhaRNA* is not. This result showed that our hierarchical strategy is capable of identifying genes that are involved in a specific phenotype.

## Discussion

Our strategy provides a method for discovering new phenotypes that is an alternative to the traditional one-on-one RNAi experiments. The analysis of the mutant collection of model organisms revealed that single-gene knockouts did not usually exhibit an observable phenotype [57-59]. Our strategy facilitates the more efficient screening of single genes that are involved in a phenotype and the simultaneous targeting of several genes; this simultaneous targeting provides an opportunity to observe additional phenotypes that can be observed only when multiple genes are silenced.

Although an siRNA can target its complementary genes, the number of mismatches that are allowed between the siRNA and its target gene(s) has not been strictly defined. According to our experimental results, at least 4 mismatches are allowed between the siRNA and its target gene(s) (Figure 4). Besides, previous reports have indicated that the targeting of the siRNA to the RNA could be affected by three, four or even five mismatches [13,50]. Therefore, using a threshold number of mismatches to distinguish the target and the non-target genes of an siRNA could generate a prediction error. To prevent the unexpected target genes of a certain siRNA, in our qualified siRNA model, two user-defined parameters, *d*_*T*_ and *d*_*N*_, were employed to predict the target and non-target genes of the qualified siRNAs, respectively (the genes within a *d*_*T*_ distance of the siRNA are its targets, whereas the genes that have at least a *d*_*N*_ distance to the siRNA are its non-targets). Theoretically, a smaller *d*_*T*_ / greater *d*_*N*_ should benefit the accuracy of the prediction of target/non-target genes because the probability of being a target gene tends to increase as the number of mismatches decreases. Nevertheless, according to our experimental results for the *Orchid* EST data (Table 2), a smaller *d*_*T*_ / greater *d*_*N*_ decreased the number of non-redundant qualified siRNAs and limited the reduction of the RNAi experiments. When the pairings of *d*_*T*_ and *d*_*N*_ were designed differently, they affected the number of RNAi experiments that would be required to screen the entire genome; however, as long as the *d*_*T*_ is set within a reasonable range (*d*_*T*_ < 5), each candidate-gene will be examined by at least one qualified siRNA.

In addition, our strategy allows the user to design two disjoint sets of genes, which are the candidate-gene set and the excluded-gene set. After that researchers narrow down the genes that could be associated with a specific phenotype into a set of candidate-genes by utilizing existing knowledge or an experimental analysis; this design can help them to focus their resources on the study of these candidate-genes. However, if a user cannot exclude any genes in advance or wants to perform a whole-genome analysis, our heuristic algorithm and hierarchical strategy can still assist in the reduction of the number of RNAi experiments that are required to perform a whole-genome analysis. We found that additional RNAi experiments can be eliminated when the excluded-gene set is empty. For example, for the *orchid* ESTs data, when the candidate-genes included all of the mRNA sequences and the TF sequences and no genes were excluded, our heuristic algorithm identified 3,092,859 qualified siRNAs with the parameters *L* = 21, *d*_*T*_ = 4 and *d*_*N*_ = 5. Only 1,674 RNAi experiments would be required to analyze 7,796 candidate-genes in the first-round RNAi analysis because there were no excluded-gene hit restrictions on the qualified siRNAs; therefore, more qualified siRNAs that can target more genes could be obtained.

Previous studies have indicated that both strands of a dsRNA can be loaded into the RNA-induced silencing complex (RISC), and they can both facilitate RNAi [54-56]. Therefore, whenever a mutant phenotype is observed, the target genes of the qualified siRNA and the potential target genes of the complementary strand of the qualified siRNAs must be examined in the second-round RNAi analysis. This step is necessary because our qualified siRNA was initially designed to be complementary to the candidate-genes. According to our analysis, the average number of possible targets of the complementary strand of a qualified siRNA was 0.6511, which indicates that only a few additional genes required screening in the second-round analysis.

Schwab et al. [60] used artificial microRNAs (amiRNA) to investigate the specificity of miRNA and showed that amiRNA can target mRNAs with a few mismatches. Artificial microRNAs can knockdown single and multiple genes and thus are an effective tool for knocking down several related but not identical genes. Schwab et al. [60] developed a tool, Web MicroRNA Designer (WMD), to facilitate the design of amiRNAs for silencing of single and multiple genes. Our strategy and that by Schwab et al. [60] are similar in that they both design small RNA to target single or multiple genes. However, WMD allows users to design effective amiRNA to knockdown their interested genes, whereas our strategy aims to reduce the initial experiments needed in a genome-wide screening.

Our initial screening using hpRNA-G55 allowed us to knockdown *PhaTF15* and *PhaTF21* simultaneously (Figure 5), and a second round of analysis using siRNAs specific to *PhaTF15* and *PhaTF21* individually indicated that TF21, similar to the previously identified transcription factor TF15, is involved in plant disease resistance (Figure 6). Interestingly, the alignment of TF15 and TF21 revealed that these two TFs share a conserved region of approximately 180 nucleotides (see Additional file 1: Figure S2 for more detail). We also found that this conserved region contains a zinc finger AN1 domain, and the target region of siRNA-G55 is located in the region of this domain. To determine whether siRNA-G55 could silence all of the genes that contain the zinc finger AN1 domain, we queried the SWISS-PROT database for all of the genes of *Arabidopsis thaliana* that contain the zinc finger AN1 domain*.* Seventeen genes were reported to contain this domain, and only two of these seventeen genes may be affected by the siRNA-G55 (when set at *d*_*T*_ = 4). It will be interesting to further determine their roles in the plant defense response.

## Conclusions

In this study, we developed a strategy to minimize the efforts required to perform high-throughput RNAi screening to identify genes involved in a specific phenotype. Our strategy took advantage of the biological phenomenon of the off-target effect of RNAi and utilized the set cover algorithm and the group testing method. We also provided a heuristic algorithm to design the siRNAs that can target multiple genes for our strategy. In the case study using the *Orchid* ESTs library, our computational result showed that 94 qualified siRNAs are sufficient to screen all 229 candidate-genes in the first-round analysis, where 229 siRNAs are required in traditional one-on-one RNAi screening. Furthermore, we verified our strategy with a previously reported transcription factor (TF15) involved in plant defense responses. In the first-round analysis, we used siRNA-G55, which is one of the 94 selected siRNAs, to silence TF15 and TF21 simultaneously, and the experimental data showed that the RNA levels of *PhaPR1* and *PhaNPR1* were subsequently affected. Moreover, in the second-round analysis, we successfully confirmed that TF15 is involved in plant defense responses only with three additional RNAi experiments. Additionally, we also indicated that TF21 is a previously unidentified transcription factor that is involved in plant defense responses.

## Abbreviations

RNAi: RNA interference; dsRNA: double-stranded RNA; RISC: RNA-induced silencing complex; TF: Transcription factor; ESTs: Expressed sequence tags; DSSP: Distinguishing substring selection problem; MMLV: Moloney murine leukemia virus; amiRNA: Artificial microRNA.

## Competing interests

The authors declare that they have no competing interests

## Authors’ contributions

CHC designed and implemented the algorithms for the analysis, performed the computational analysis, and drafted the paper. HHY and HIW conceived of the study, participated in the design and drafted the paper. HHY, HCL and CEC performed the wet experiments. HHC provided the transcription factor sequence data. HHY and CYT provided constructive opinions and refined the manuscript. All authors read and approved the final manuscript.

## Supplementary Material

Additional file 1 **Figures S1 and S2.** A simple example of a powerful subsequence *P*, and the dotplot of TF15 and TF21 sequences. Click here for file

Additional file 2 **Proof to prove that the problem of finding a qualified sequence *r *maximizing the size of *T *is NP-complete.** Click here for file

Additional file 3 Methods the detail of our modified greedy algorithm for selecting qualified siRNAs to perform the first-round RNAi analysis. Click here for file

Additional file 4 ** Tables S1-3.** The lists of the siRNAs that were selected for first-round RNAi analysis with different parameters. Click here for file

## References

[B1] Bennetzen JL Kellogg EA Lee M Messing J A plant genome initiative Plant Cell 1998 10 488 493

[B2] Dong Y Burch-Smith TM Liu Y Mamillapalli P Dinesh-Kumar SP A ligation-independent cloning tobacco rattle virus vector for high-throughput virus-induced gene silencing identifies roles for NbMADS4-1 and -2 in floral development Plant Physiol 2007 145 1161 1170 10.1104/pp.107.107391 17932306 PMC2151726

[B3] Wilson RK Schnable PS Ware D Fulton RS Stein JC Wei FS Pasternak S Liang CZ Zhang JW Fulton L The B73 maize genome: complexity, diversity, and dynamics Science 2009 326 1112 1115 10.1126/science.1178534 19965430

[B4] Chandler VL Brendel V The maize genome sequencing project Plant Physiol 2002 130 1594 1597 10.1104/pp.015594 12481042 PMC1540264

[B5] Gill BS Appels R Botha-Oberholster AM Buell CR Bennetzen JL Chalhoub B Chumley F Dvorak J Iwanaga M Keller B A workshop report on wheat genome sequencing: international genome research on wheat consortium Genetics 2004 168 1087 1096 10.1534/genetics.104.034769 15514080 PMC1448818

[B6] Sasaki T Burr B International rice genome sequencing project: the effort to completely sequence the rice genome Curr Opin Plant Biol 2000 3 138 141 10.1016/S1369-5266(99)00047-3 10712951

[B7] International Rice Genome Sequencing Project The map-based sequence of the rice genome Nature 2005 436 793 800 10.1038/nature03895 16100779

[B8] Rounsley SD Last RL Shotguns and SNPs: how fast and cheap sequencing is revolutionizing plant biology Plant J 2010 61 922 927 10.1111/j.1365-313X.2009.04030.x 20409267

[B9] Metzker ML Sequencing technologies - the next generation Nat Rev Genet 2010 11 31 46 10.1038/nrg2626 19997069

[B10] Imelfort M Edwards D De novo sequencing of plant genomes using second-generation technologies Brief Bioinform 2009 10 609 618 10.1093/bib/bbp039 19933209

[B11] Liu J Huang S Peng X Liu W Wang H Studies on high salt tolerance of transgenic tobacco Chin J Biotechnol 1995 11 275 280 8739106

[B12] Abebe T Guenzi AC Martin B Cushman JC Tolerance of mannitol-accumulating transgenic wheat to water stress and salinity Plant Physiol 2003 131 1748 1755 10.1104/pp.102.003616 12692333 PMC166930

[B13] Ossowski S Schwab R Weigel D Gene silencing in plants using artificial microRNAs and other small RNAs Plant J 2008 53 674 690 10.1111/j.1365-313X.2007.03328.x 18269576

[B14] Fire A Xu S Montgomery MK Kostas SA Driver SE Mello CC Potent and specific genetic interference by double-stranded RNA in Caenorhabditis elegans Nature 1998 391 806 811 10.1038/35888 9486653

[B15] Keating CD Kriek N Daniels M Ashcroft NR Hopper NA Siney EJ Holden-Dye L Burke JF Whole-genome analysis of 60 G protein-coupled receptors in Caenorhabditis elegans by gene knockout with RNAi Curr Biol 2003 13 1715 1720 10.1016/j.cub.2003.09.003 14521838

[B16] Sugimoto A High-throughput RNAi in Caenorhabditis elegans: genome-wide screens and functional genomics Differentiation 2004 72 81 91 10.1111/j.1432-0436.2004.07202004.x 15066188

[B17] Agaisse H Burrack LS Philips JA Rubin EJ Perrimon N Higgins DE Genome-wide RNAi screen for host factors required for intracellular bacterial infection Science 2005 309 1248 1251 10.1126/science.1116008 16020693

[B18] Furlong EE A functional genomics approach to identify new regulators of Wnt signaling Dev Cell 2005 8 624 626 10.1016/j.devcel.2005.04.006 15866154

[B19] McGinnis K Chandler V Cone K Kaeppler H Kaeppler S Kerschen A Pikaard C Richards E Sidorenko L Smith T Transgene-induced RNA interference as a tool for plant functional genomics Methods Enzymol 2005 392 1 24 15644172 10.1016/S0076-6879(04)92001-0

[B20] Hilson P Allemeersch J Altmann T Aubourg S Avon A Beynon J Bhalerao RP Bitton F Caboche M Cannoot B Versatile gene-specific sequence tags for Arabidopsis functional genomics: transcript profiling and reverse genetics applications Genome Res 2004 14 2176 2189 10.1101/gr.2544504 15489341 PMC528935

[B21] Dietzl G Chen D Schnorrer F Su KC Barinova Y Fellner M Gasser B Kinsey K Oppel S Scheiblauer S A genome-wide transgenic RNAi library for conditional gene inactivation in Drosophila Nature 2007 448 151 156 10.1038/nature05954 17625558

[B22] Yang JP Fan W Rogers C Chatterton JE Bliesath J Liu G Ke N Wang CY Rhoades K Wong-Staal F Li QX A novel RNAi library based on partially randomized consensus sequences of nuclear receptors: identifying the receptors involved in amyloid beta degradation Genomics 2006 88 282 292 10.1016/j.ygeno.2006.03.010 16631344

[B23] Moffat J Grueneberg DA Yang X Kim SY Kloepfer AM Hinkle G Piqani B Eisenhaure TM Luo B Grenier JK A lentiviral RNAi library for human and mouse genes applied to an arrayed viral high-content screen Cell 2006 124 1283 1298 10.1016/j.cell.2006.01.040 16564017

[B24] International Human Genome Sequencing Consortium Finishing the euchromatic sequence of the human genome Nature 2004 431 931 945 10.1038/nature03001 15496913

[B25] The Arabidopsis Genome Initiative Analysis of the genome sequence of the flowering plant Arabidopsis thaliana Nature 2000 408 796 815 10.1038/35048692 11130711

[B26] Sun DQ Melegari M Sridhar S Rogler CE Zhu L Multi-miRNA hairpin method that improves gene knockdown efficiency and provides linked multi-gene knockdown Biotechniques 2006 41 59 63 10.2144/000112203 16869514

[B27] Xia XG Zhou HX Xu ZS Multiple shRNAs expressed by an inducible pol II promoter can knock down the expression of multiple target genes Biotechniques 2006 41 64 68 10.2144/000112198 16869515

[B28] Ouyang B Yang T Li H Zhang L Zhang Y Zhang J Fei Z Ye Z Identification of early salt stress response genes in tomato root by suppression subtractive hybridization and microarray analysis J Exp Bot 2007 58 507 520 17210988 10.1093/jxb/erl258

[B29] Liu HH Tian X Li YJ Wu CA Zheng CC Microarray-based analysis of stress-regulated microRNAs in Arabidopsis thaliana RNA 2008 14 836 843 10.1261/rna.895308 18356539 PMC2327369

[B30] Jackson AL Bartz SR Schelter J Kobayashi SV Burchard J Mao M Li B Cavet G Linsley PS Expression profiling reveals off-target gene regulation by RNAi Nat Biotechnol 2003 21 635 637 10.1038/nbt831 12754523

[B31] Moffat J Reiling JH Sabatini DM Off-target effects associated with long dsRNAs in Drosophila RNAi screens Trends Pharmacol Sci 2007 28 149 151 10.1016/j.tips.2007.02.009 17350110

[B32] Kulkarni MM Booker M Silver SJ Friedman A Hong P Perrimon N Mathey-Prevot B Evidence of off-target effects associated with long dsRNAs in Drosophila melanogaster cell-based assays Nat Methods 2006 3 833 838 16964256 10.1038/nmeth935

[B33] Du D Hwang F Combinatorial group testing and its applications 2000 2 Singapore: World Scientific

[B34] Tsai WC Hsiao YY Lee SH Tung CW Wang DP Wang HC Chen WH Chen HH Expression analysis of the ESTs derived from the flower buds of Phalaenopsis equestris Plant Sci 2006 170 426 432 10.1016/j.plantsci.2005.08.029

[B35] Altschul SF Gish W Miller W Myers EW Lipman DJ Basic local alignment search tool J Mol Biol 1990 215 403 410 2231712 10.1016/S0022-2836(05)80360-2

[B36] Fu CH Chen YW Hsiao YY Pan ZJ Liu ZJ Huang YM Tsai WC Chen HH OrchidBase: a collection of sequences of the transcriptome derived from orchids Plant Cell Physiol 2011 52 238 243 10.1093/pcp/pcq201 21245031

[B37] Bartel DP MicroRNAs: genomics, biogenesis, mechanism, and function Cell 2004 116 281 297 10.1016/S0092-8674(04)00045-5 14744438

[B38] Schwab R Palatnik JF Riester M Schommer C Schmid M Weigel D Specific effects of microRNAs on the plant transcriptome Dev Cell 2005 8 517 527 10.1016/j.devcel.2005.01.018 15809034

[B39] Haley B Zamore PD Kinetic analysis of the RNAi enzyme complex Nat Struct Mol Biol 2004 11 599 606 10.1038/nsmb780 15170178

[B40] Reynolds A Leake D Boese Q Scaringe S Marshall WS Khvorova A Rational siRNA design for RNA interference Nat Biotechnol 2004 22 326 330 10.1038/nbt936 14758366

[B41] Hsieh AC Bo RH Manola J Vazquez F Bare O Khvorova A Scaringe S Sellers WR A library of siRNA duplexes targeting the phosphoinositide 3-kinase pathway: determinants of gene silencing for use in cell-based screens Nucleic Acids Res 2004 32 893 901 10.1093/nar/gkh238 14769947 PMC373385

[B42] Takasaki S Kotani S Konagaya A An effective method for selecting siRNA target sequences in mammalian cells Cell Cycle 2004 3 790 795 15118413

[B43] Karimi M Inze D Depicker A GATEWAY vectors for Agrobacterium-mediated plant transformation Trends Plant Sci 2002 7 193 195 10.1016/S1360-1385(02)02251-3 11992820

[B44] Tian T Klaassen VA Soong J Wisler G Duffus JE Falk BW Generation of cDNAs specific to lettuce infectious yellows closterovirus and other whitefly-transmitted viruses by RT-PCR and degenerate oligonucleotide primers corresponding to the closterovirus gene encoding the heat shock protein 70 homolog Phytopathology 1996 86 1167 1173 10.1094/Phyto-86-1167

[B45] Lu HC Chen HH Tsai WC Chen WH Su HJ Chang DC Yeh HH Strategies for functional validation of genes involved in reproductive stages of orchids Plant Physiol 2007 143 558 569 17189336 10.1104/pp.106.092742 PMC1803755

[B46] Lu HC Hsieh MH Chen CE Chen HH Wang HI Yeh HH A high-throughput virus-induced gene-silencing vector for screening transcription factors in virus-induced plant defense response in orchid Mol Plant Microbe Interact 2012 25 738 746 10.1094/MPMI-10-11-0266 22397405

[B47] Deng X Li G Li Z Ma B Wang LS Genetic design of drugs without side-effects SIAM J Comput 2003 32 1073 1090 10.1137/S0097539701397825

[B48] Lanctot JK Li M Ma B Wang SJ Zhang LX Distinguishing string selection problems Inform and Comput 2003 185 41 55 10.1016/S0890-5401(03)00057-9

[B49] Tang G Reinhart BJ Bartel DP Zamore PD A biochemical framework for RNA silencing in plants Genes Dev 2003 17 49 63 10.1101/gad.1048103 12514099 PMC195971

[B50] Palatnik JF Allen E Wu X Schommer C Schwab R Carrington JC Weigel D Control of leaf morphogenesis by microRNAs Nature 2003 425 257 263 10.1038/nature01958 12931144

[B51] Garey MR Johnson DS Computers and intractability: a guide to the theory of NP-completeness 1979 San Francisco: W. H. Freeman

[B52] Lovasz L Ratio of Optimal Integral and Fractional Covers Discrete Math 1975 13 383 390 10.1016/0012-365X(75)90058-8

[B53] Johnson DS Approximation Algorithms for Combinatorial Problems J Comput System Sci 1974 9 256 278 10.1016/S0022-0000(74)80044-9

[B54] Elbashir SM Lendeckel W Tuschl T RNA interference is mediated by 21- and 22-nucleotide RNAs Genes Dev 2001 15 188 200 10.1101/gad.862301 11157775 PMC312613

[B55] Elbashir SM Martinez J Patkaniowska A Lendeckel W Tuschl T Functional anatomy of siRNAs for mediating efficient RNAi in Drosophila melanogaster embryo lysate EMBO J 2001 20 6877 6888 10.1093/emboj/20.23.6877 11726523 PMC125328

[B56] Nykanen A Haley B Zamore PD ATP requirements and small interfering RNA structure in the RNA interference pathway Cell 2001 107 309 321 10.1016/S0092-8674(01)00547-5 11701122

[B57] Hanada K Kuromori T Myouga F Toyoda T Li WH Shinozaki K Evolutionary persistence of functional compensation by duplicate genes in Arabidopsis Genome Biol Evol 2009 1 409 414 20333209 10.1093/gbe/evp043 PMC2817435

[B58] Conant GC Wagner A Duplicate genes and robustness to transient gene knock-downs in Caenorhabditis elegans Proc Biol Sci 2004 271 89 96 10.1098/rspb.2003.2560 15002776 PMC1691561

[B59] Bouche N Bouchez D Arabidopsis gene knockout: phenotypes wanted Curr Opin Plant Biol 2001 4 111 117 10.1016/S1369-5266(00)00145-X 11228432

[B60] Schwab R Ossowski S Riester M Warthmann N Weigel D Highly specific gene silencing by artificial microRNAs in Arabidopsis Plant Cell 2006 18 1121 1133 10.1105/tpc.105.039834 16531494 PMC1456875

