# Supplementary material for: An efficient RNA interference screening strategy for gene functional analysis
Source: BMC Genomics. 2012 Sep 18;13:491. doi: 10.1186/1471-2164-13-491 (PMC3533828; doi:10.1186/1471-2164-13-491)
Supplement: Additional file 1 — Figures S1 and S2. A simple example of a powerful subsequence P, and the dotplot of TF15 and TF21 sequences. [file 1471-2164-13-491-S1.pdf]

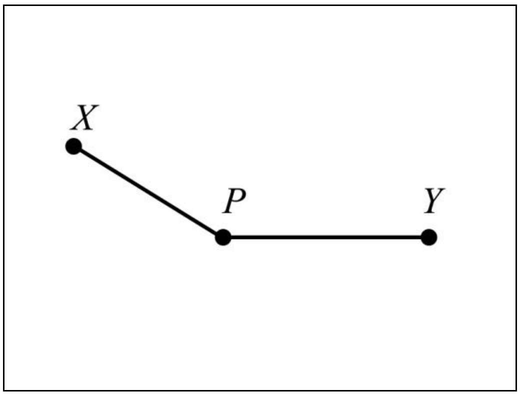

Figure S1. A simple example of a powerful subsequence *P*. *X*, *Y* and *P* are subsequences, and the edges indicate the neighborhood relationship of these variables. According to this figure, *X* and *Y* are the ***neighbors*** of *P*, but *X* and *Y* are not close to each other. In the case, the reverse complement sequence of *P* has the potential to silence all three of the genes (*X*, *Y* and *P*), but the reverse complement sequence of *X* or *Y* can only silence two of the genes. Because *P* can cover all of its *neighbors*' target genes, *P* is more powerful than *X* and *Y*. In this case, we say *P* is a powerful subsequence, and *X* and *Y* are dominated by *P*.

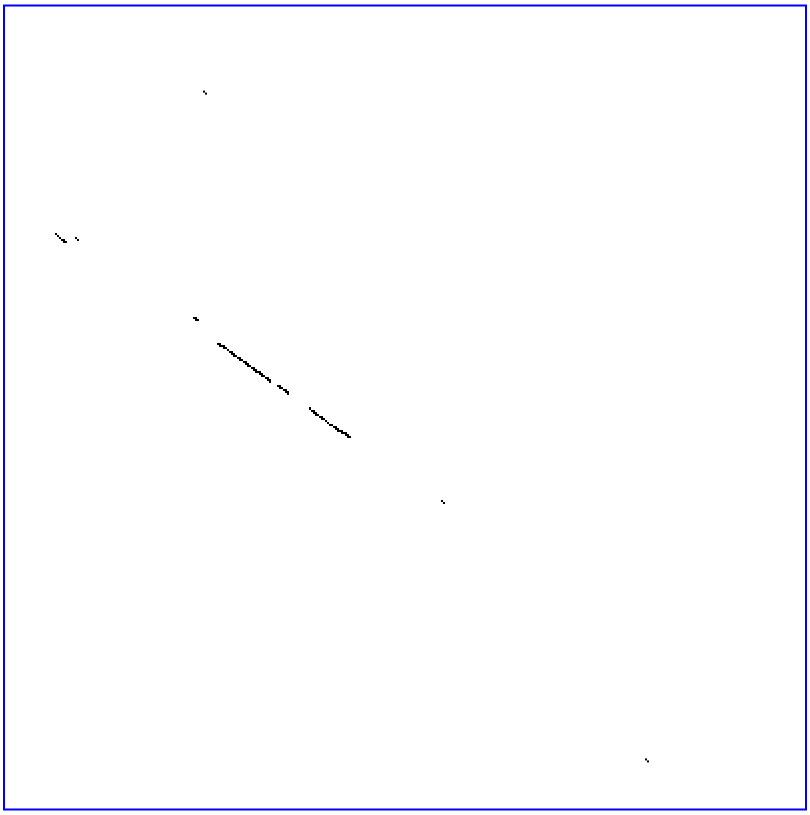

Figure S2. The dotplot of TF15 and TF21 sequences with parameters window size and mismatch set as 21 and 5, respectively.
