# Supplementary material for: An efficient RNA interference screening strategy for gene functional analysis
Source: BMC Genomics. 2012 Sep 18;13:491. doi: 10.1186/1471-2164-13-491 (PMC3533828; doi:10.1186/1471-2164-13-491)
Supplement: Additional file 2 — Proof to prove that the problem of finding a qualified sequence r maximizing the size of T is NP-complete. [file 1471-2164-13-491-S2.pdf]

The *distinguishing substring selection* problem (DSSP) has been proven to be NP-complete, and it is defined as follows:

Input:

- A set $S_g = \{s'_1, s'_2, \ldots, s'_{n1}\}$ of n_1 (good) strings of length at least L.
- A set $S_b = \{s_1, s_2, \ldots, s_{n2}\}$ of n_2 (bad) strings of length at least L.
- Two non-negative integers d_g and d_b, $d_g > d_b$.

Output:

Find a string x such that each string $s_i \in S_b$ has at least a length L substring v_i of s_i with $HD(x, v_i) \leq d_b$, and for every string $s'_i \in S_g$, $HD(x, v'_i) \geq d_g$ for each length-L substring v'_i of s'_i.

The *qualified sequence finding problem* is defined as follows:

Input:

Given a set of n candidate-genes, $C = \{c_1, c_2, \ldots, c_n\}$, a set of m excluded-genes, $E = \{e_1, e_2, \ldots, e_m\}$, and three integers L, d_N and d_T, $L > d_N > d_T$.

Output:

Find a sequence r of length L such that there exists a subset of candidate-genes T, $T \subseteq C$, and for each gene $t_i \in T$, $HD(r, u_i) \leq d_T$ for some length L substring u_i of t_i and for each gene $g_i \in \{E \cup C - T\}$, $HD(r, u'_i) \geq d_N$ for any length L substring u'_i of g_i.

Theorem 1. The problem of finding a qualified sequence r maximizing the size of T is NP-complete.

Proof.

1. Finding a qualified sequence r maximizing the size of T is in NP.

Given a qualified sequence r, we can verify the sequence and obtain the size of T in polynomial time. We compute the Hamming distance between r and all subsequences, which are enumerated from the candidate-genes and the excluded-genes by a sliding window. Next, we examine whether r is within a d_T distance to some candidate-gene(s) and has at least a d_N distance to all of the other genes. If r meets the above conditions, then r is valid, and the size of T is the number of candidate-genes that are within d_T distance to r. Otherwise, r is invalid, and the size of T is zero.

2. Reduce the DSSP to the problem of finding a qualified sequence r maximizing the size of T.

Given an instance I of DSSP, we construct an instance F of finding a qualified sequence r maximizing the size of T as follows. Let $C=S_b$, $E=S_g$, $d_N=d_g$ and $d_T=d_b$. Obviously, this can be done in polynomial time. Now, we show that there is a string x that meets the conditions for instance I if and only if there is a valid sequence r maximizing the size of T and $T = C$ for instance F.

First, suppose there is a string x such that each string $s_i \in S_b$ has at least a length L substring v_i of s_i with $HD(x, v_i) \le d_b$ and in every string $s'_i \in S_g$ for each length L substring v'_i of s'_i, $HD(x, v'_i) \ge d_g$. Define sequence $r = x$ and $T = S_b$. Obviously, each sequence $t_i \in T$ has a length L substring u_i of t_i with $HD(r, u_i) \le d_T$ in every sequence $g_i \in \{E \cup C - T\}$ for each length L substring u'_i of g_i, $HD(r, u'_i) \ge d_N$, and the size of T is maximum.

Conversely, suppose there is a sequence r and T, where $T = C$ such that each string $t_i \in T$ has a length L substring u_i of t_i with $HD(r, u_i) \le d_T$, and for every string $g_i \in \{E \cup C - T\}$, $HD(r, u'_i) \ge d_N$ for each length L substring u'_i of g_i. Define string $x = r$. Obviously, each string $s_i \in S_b$ has a length L substring v_i of s_i with

$HD(x, v_i) \leq d_b$ and for every string $s'_i \in S_g$, for any length-L substring v'_i of s'_i, $HD(x, v'_i) \geq d_g$.

Because DSSP can be reduced to the problem of finding a qualified sequence r maximizing the size of T in polynomial time, finding a qualified sequence r maximizing the size of T is an NP-complete problem.
