# Supplementary material for: An efficient RNA interference screening strategy for gene functional analysis
Source: BMC Genomics. 2012 Sep 18;13:491. doi: 10.1186/1471-2164-13-491 (PMC3533828; doi:10.1186/1471-2164-13-491)
Supplement: Additional file 3 — Methods the detail of our modified greedy algorithm for selecting qualified siRNAs to perform the first-round RNAi analysis. [file 1471-2164-13-491-S3.pdf]

Greedy algorithm for set cover

We adopted the original greedy algorithm for the set cover problem (Johnson, 1974; Lovasz, 1975) and slightly modified it to increase the examination frequency of each candidate-gene. The modification was that at each step, we selected the unused siRNAs that could target the largest number of uncovered candidate-genes and also had the largest number of target genes. This modification allowed us to increase the average examination frequency of the candidate-genes. The approximation ratio of this modified algorithm is still H_k, where $H_k = \sum_{i=1}^{k} \frac{1}{i}$ and k is the size of the largest set. In practice, we can repeat this greedy algorithm many times, and each time, the order of the qualified siRNAs and their target genes was shuffled to provide a different solution. Then, we chose the solution that displayed the smallest size from all of the solutions as the output. Here, we describe our modified greedy algorithm for selecting qualified siRNAs to perform the first round of RNAi analysis. We first give the definition of the problem.

Input :

$C = \{c_1, c_2, ..., c_n\}$: the set of candidate genes;

$R = \{r_1, r_2, ..., r_m\}$: the set of qualified siRNAs;

$Target = \{T_1, T_2, ..., T_m\}$: the set of target gene set of each qualified siRNA $r_j, 1 \le j \le m$

$Rpeated\_num$: The repeating number of the for loop.

Output :

A set of siRNAs such that the collection of these siRNAs' target genes covers C

Our modified greedy algorithm works as follows:

for $k = 1$ to $Rpeated\_num$\{
    $Pool = C$;
    $Q = \{q_1, q_2, ..., q_m\} = Target$;
    $siRNA\_for\_use = \phi$;
    while ($Pool \neq \phi$)\{
        Choose a smaller subscript j such that (q_j, T_j) is maximized
        $Temp = Temp \cup \{r_j\}$;
        $Pool = Pool - T_j$;
        foreach $q_i \in Q, q_i = q_i - q_j$;
    \}
    if (($|Temp| < |siRNA\_set|$) or ($siRNA\_set == \phi$))\{
        $siRNA\_set = Temp$;
    \}
    Shuffle the order of R and $Target$ wtihout loss of their corresponding relationship;
\}
return $siRNA\_set$;
