# Supplementary material for: An efficient RNA interference screening strategy for gene functional analysis
Source: BMC Genomics. 2012 Sep 18;13:491. doi: 10.1186/1471-2164-13-491 (PMC3533828; doi:10.1186/1471-2164-13-491)
Supplement: Additional file 4 — Tables S1-3. The lists of the siRNAs that were selected for first-round RNAi analysis with different parameters. [file 1471-2164-13-491-S4.pdf]

Table S1

Parameters: d_T=4, d_N=5, L=21

Number of non-redundant qualified siRNAs: **2147**

Number of qualified siRNAs selected in the first round of RNAi analysis: **94**

Number of candidate-genes examined in the first round of RNAi analysis: **229**

Average examination frequency of each gene: **1.17030567686**

No.	Selected qualified siRNA	Target genes of qualified siRNA (TF number)	*Qualified sequence		**Mismatch numbers
			TF number	Position	
1	UCUCAAUCUUCUUUAUCUGCA	209, 207, 210, 208, 68, 86, 45, 58	58	75	4, 4, 3, 2, 4, 4, 4, 0
2	UUCCCCUCCCCGACAUCUCCU	206, 75, 108, 23, 116	116	37	4, 4, 4, 4, 0
3	AUCCGCCGUCGCCGCCGUCUC	4, 25, 106, 61, 49	49	322	4, 4, 4, 3, 0
4	CCAUCAGCCGCUGCUCCAGCG	158, 37, 6, 120	120	350	4, 4, 4, 0
5	AAGGUGCCAAGCCAAGCCCGG	149, 102, 112, 90	90	240	4, 4, 2, 0
6	CUUUAACCUUCUUCUGGAUUC	220, 132, 36, 221	221	443	2, 4, 2, 0
7	ACAUAGUUUAUAAUCUCAUCG	159, 73, 67, 146	146	158	4, 4, 4, 0
8	GACAAGGUGCUUGUUAUUGUU	217, 222, 39, 70	70	359	4, 2, 4, 0
9	AUAAAAAGUUACGGUUGAAGU	167, 175, 82, 74	74	254	4, 4, 4, 0
10	UACAAAAUAUAUAUAUGAGCA	38, 77, 3, 145	145	874	4, 4, 0, 0
11	AAUCAUAAUUUAAGGUUCCAA	120, 97, 47, 5	5	871	2, 4, 4, 0
12	GCGAGAACAUGAGUAGCAAAA	209, 66, 29, 60	60	214	4, 4, 4, 0
13	CACCUGCGGCGCCCAUCCCCU	125, 215, 50, 217	217	1018	4, 4, 4, 0
14	GUAAAGGAAAACAACAGAGAA	207, 165, 200, 85	85	352	4, 4, 4, 0
15	UUCCAACUUCAGUUAAUUCUG	174, 26, 1	1	209	4, 4, 0
16	CAGCCAAACUUGUCUAGCAAC	103, 32, 79	79	1035	4, 4, 0
17	UCACUUUCGUCUAUAUUCAUC	118, 183, 194	194	487	4, 3, 0
18	CAGAAUGGCAGCUUCAUCAUG	62, 65, 35	35	326	4, 4, 0
19	UCACCAGUAUCUGAAAAGCCA	135, 98, 163	163	286	4, 4, 0
20	GAAAGCUGAGGCCUUUGCCGC	87, 76, 197	197	264	4, 4, 0
21	CUUCCAGCUCCUCUUCUCCGC	148, 124, 43	43	768	4, 4, 0
22	CCAAUCAAAGAACUAAAUGUC	100, 83, 219	219	1365	4, 4, 0
23	CAUCUCAUCGAGUAUGAAAAG	171, 31, 212	212	1220	3, 4, 0
24	AAUCAUUGGCUACGGGGCAUU	170, 138, 127	127	158	4, 3, 0
25	CCGGGGGAAGUGGCGGCAGAG	227, 192, 199	199	696	4, 3, 0
26	CGCGGCAGCGGCAGCCACCGU	218, 30, 78	78	770	4, 3, 0
27	CAGUGAUCAGAGCAAUACCAG	201, 27, 72	72	742	4, 4, 0
28	CACACUCCCUCCUCCCUGCAU	224, 9, 164	164	825	4, 4, 0
29	UCAGUUGGAUGGAAUCGGAAU	95, 33, 155	155	276	4, 3, 0
30	UGUGUGCUAUGUCUGGAAAUG	133, 91, 71	71	1748	4, 4, 0
31	CGUAGCGUAUUGUUUUCUCAA	223, 28, 12	12	24	3, 4, 0

**32**	AAAACAACAUAAGGAUCACUG	188, 2, 48	48	805	3, 4, 0
**33**	AAACUGCAUUUUCCUCCUUCA	130, 166, 162	162	156	4, 4, 0
**34**	AUUUUCUGCAUCCAUGGAAAU	131, 151, 96	96	883	4, 4, 0
**35**	UUGCAACAAAAAAUUAAGAUC	123, 10, 172	172	408	4, 4, 0
**36**	UGUAGCUUUUCCAAUCCUCAA	154, 160, 156	156	326	4, 4, 0
**37**	AAAACAGAACUUACCUGUUCC	111, 11, 63	63	1206	4, 4, 0
**38**	UAUAAAACUCAUAUAUCUUUC	129, 211, 134	134	1021	4, 4, 0
**39**	CUCUCGGCUUCCACAUGGUUC	205, 189, 190	190	1	3, 4, 0
**40**	AUACCUCCACCGUUCAAAGCA	176, 77, 57	57	147	4, 2, 0
**41**	UCUGCCCAUAUUUCCUCCACU	187, 106, 157	157	502	2, 4, 0
**42**	GGCCAAGAGCUCGUCUUCCUC	80, 106, 69	69	114	4, 4, 0
**43**	AACACGAAGCAAAAUACAGUA	47, 22, 119	119	230	4, 4, 0
**44**	ACAUCCAUGCACGAAGAACAG	32, 18, 204	204	514	4, 4, 0
**45**	AAUUGGACGGCUGGAUUUGAA	150, 144, 192	192	109	4, 4, 0
**46**	UUUUCAGCUCCAGUGAUCAGA	198, 41, 72	72	752	4, 3, 0
**47**	ACAAAUUAAUAGAAAACGGAA	210, 153, 99	99	507	4, 4, 0
**48**	CAUUUUAUAUUUUUUUCAAUU	53, 50, 46	46	822	4, 4, 0
**49**	ACUCCAUAACUAAAUCAAACU	213, 55, 73	73	715	4, 4, 0
**50**	AUACAUAUCUCAUAAUCCAAA	142, 56, 224	224	259	4, 3, 0
**51**	AAUUACUCCCACAAAGCAAAG	147, 39, 59	59	236	4, 4, 0
**52**	AAUGAAAAUCUGUAACACAUC	122, 227, 89	89	1028	4, 4, 0
**53**	AAACAUAAAAUUAGAGAACAG	107, 109, 63	63	1485	4, 3, 0
**54**	CAUUGUGGUUCAGAAUCCUCA	101, 8	8	167	4, 0
**55**	CGUGCUGCUCCGGGUACCGGU	15, 21	21	418	1, 0
**56**	AAACAAAAAAAUAUCCUUUCG	113, 139	139	227	4, 0
**57**	CCAGUUUGAAACCUGGAAGAA	117, 143	143	303	4, 0
**58**	UGUUUCCCAACCCUACCAAAC	94, 44	44	306	4, 0
**59**	CUGGAAUUUAAAGUGUGUUGC	136, 177	177	85	4, 0
**60**	GGAGUUUGCACAUUGCUCAAU	40, 93	93	328	4, 0
**61**	AAUUUCAGUUUGCAUGAAUGU	34, 181	181	287	4, 0
**62**	UUGCAGUGCCCACACUUCACA	179, 52	52	78	4, 0
**63**	AUGUUGUCUCCUCCUUCCAUA	228, 216	216	940	4, 0
**64**	AAAUCCCGGCGGCAAGUUCAG	137, 169	169	82	4, 0
**65**	CAUCUCGCUUCUGAAUUUGAU	121, 229	229	979	4, 0
**66**	GAUAACAGUUUUUUCAAAUUU	92, 152	152	78	4, 0
**67**	CUGAGAGCUAGAACAAAAUUA	180, 226	226	11	4, 0
**68**	AUGGAAGCAUGAUACAAAAGC	19, 110	110	679	4, 0
**69**	CUCCAUGGAGAAAGAGAGCUA	161, 20	20	540	4, 0
**70**	GGUAAAUGAAAACCCUGUGGA	193, 24	24	421	4, 0
**71**	UAUAUCCUCCUCCAUACCUUU	214, 186	186	161	4, 0
**72**	AUUAGCUCCUUUGCUCAAUCC	64, 54	54	458	4, 0

**73**	AAACAGCUAAAUUGCAGUACG	81, 225	225	1281	4, 0
**74**	GUACUUCCCCGAUGAUGCCGC	140, 51	51	485	4, 0
**75**	CCCCCCGCCAUGAAGAGGACC	13, 141	141	560	4, 0
**76**	AGGAAAAUUGAGGUUUUUCAA	114, 115	115	66	4, 0
**77**	CAGCCUCGUCAGCGCAGCAGA	195, 168	168	160	4, 0
**78**	ACGCGGAGGAGUAAAAUCGGC	128, 202	202	161	4, 0
**79**	CAGCCAGAAGCUUGAUUUGUU	173, 196	196	164	4, 0
**80**	ACAGCUAAAAUGACCUUCCUA	17, 104	104	254	4, 0
**81**	AGAUCCAUGAUCAACAACUGC	220, 184, 221	221	333	2, 4, 0
**82**	UCUUCCCCCUGGCCAUCUACA	206, 209, 14	14	167	4, 3, 0
**83**	AGGAGCCCCACCUUCUUCCGG	191, 15, 21	21	356	4, 4, 0
**84**	AAUGCCAGAUACCUGCUGGUA	203, 208, 209	209	418	4, 3, 0
**85**	AAAAAAUUGCCUUCUUGCAUG	88, 86, 45	45	838	4, 1, 0
**86**	AGUAAGCCUUGAUUGGCAGAA	105, 91, 95	95	856	4, 4, 0
**87**	UAUUUCAGUGGUCACCAUGUC	126, 77, 57	57	611	4, 2, 0
**88**	AGAGCUCCGGCGAGAGUUUCG	53, 7	7	395	4, 0
**89**	GGAUCACGUGUCAAAGCCAUU	123, 42	42	401	4, 0
**90**	AUCCGUCCUAAGAAACAAGAA	164, 182	182	113	4, 0
**91**	CUGCUCCUUCUUCCAAGCAUC	199, 16	16	105	4, 0
**92**	GAACGCGUAUGACAUGCAGAC	185, 227	227	848	4, 0
**93**	CCUAGACAACCAGUGAAACUC	177, 178	178	131	3, 0
**94**	AAAAAGUAUUACCUUUUAACA	84, 212	212	1530	4, 0

*** The Transcription factor numbers and postions of the qualified sequences which the selected qualified siRNAs are derived from.**

**** The mismatch numbers between qualified siRNA and its target genes.**

Table S2

Parameters: d_T**=4,** d_N**=6,** L**=21**

Number of non-redundant qualified siRNAs: **352**

Number of qualified siRNAs selected in the first round of RNAi analysis: **162**

Number of candidate-genes examined in the first round of RNAi analysis: **227**

Average examination frequency of each gene: **1.11013215859**

No.	Selected qualified siRNA	Target genes of qualified siRNA (TF number)	*Qualified sequence		**Mismatch numbers
			TF number	Position	
1	UAUCGUAAGCUCGGGCCGCGG	167, 112, 74, 175	175	167	2, 3, 3, 0
2	AAUAUGUAACUUGCCUGUUCG	206, 208, 210, 207	207	112	4, 4, 4, 0
3	GGGGAGUUGCCUCGCAAUUUU	221, 220, 10, 36	36	27	3, 0, 4, 0
4	GAAUGCCGCUACUCUCAUGAU	32, 45, 86	86	503	4, 2, 0
5	CGACUUGAGUUGGUGUCCUUG	120, 49, 5	5	113	1, 4, 0
6	GCUGAUUCUCGGUUGGACAGC	24, 85, 165	165	649	3, 4, 0
7	ACCUUUGAUUUAUGUAAACGA	143, 80, 48	48	1269	4, 4, 0
8	CACGGCUGCCCUAUUAGCCGC	158, 6, 173	173	279	4, 4, 0
9	AGGUCACCUGCCGAUUAGUCG	211, 75, 209	209	248	4, 4, 0
10	CGUAGCGUAUUGUUUUCUCAA	223, 28, 12	12	24	3, 4, 0
11	UCCACAGCCUAACUCUUGCGU	91, 32, 204	204	634	4, 1, 0
12	AGAUGCUCGUGCCACCUCGGA	94, 151	151	686	2, 0
13	UUGUUGGCUGCGUGGACUUCC	133, 168	168	203	4, 0
14	GGUUCCGGUGCCCGUCGCGGC	184, 30	30	343	4, 0
15	UGGGAAAAUUCAAGUCGGAUC	155, 227	227	2533	4, 0
16	GAACGGUAAUGCAUGUAAAAU	127, 170	170	559	0, 0
17	CUUGAUCACAGGAUUGCUGCG	7, 21	21	483	4, 0
18	UCUGCAUCCUAAAUUGGCAUA	122, 134	134	437	4, 0
19	UUGCAAGCCCAGAUGAGUCUC	226, 166	166	232	4, 0
20	GCUCCGCAAAGAACGGCCAAG	215, 216	216	1294	3, 0
21	AAUCUCGUACGCUAUUGAUGA	145, 63	63	562	4, 0
22	UCGAAUCUGUUUAAGAGAGGA	60, 68	68	493	4, 0
23	AGAAACUAGGCAGACUUGUCU	119, 55	55	140	4, 0
24	AGAGUAACCACUGGAUCGGAA	160, 81	81	182	4, 0
25	CGAACUCAUCCAAGCACACCG	62, 14	14	1	4, 0
26	CCCGCACAGAAUAUGAGCCCA	153, 121	121	561	4, 0
27	GCCCCUUAAUCAUGCAGAUAG	35, 25	25	624	4, 0
28	AUUCAAGAGGCUAAGCAUGGG	123, 29	29	764	4, 0
29	GUGCACCGAAAUUAACCAAAA	27, 219	219	877	4, 0
30	CUGGACGGCGGAUCUCGACUG	92, 61	61	57	4, 0
31	AAAGAGUAGACUGCUCCUUAG	37, 125	125	314	4, 0

**32**	GCCUGCACAUAGUUUAUAAUC	67, 146	146	164	4, 0
**33**	ACUCAUGCAUGAUCCAUUCAG	95, 33	33	549	4, 0
**34**	UUGGUGACCGUAACCUGUGUA	58, 79	79	332	4, 0
**35**	UCGCAAAUGGCCUCCAGCCUA	90, 102	102	945	1, 0
**36**	CGGCGUGUCACCCCUGUACAU	43, 148	148	208	4, 0
**37**	ACUUACAAGUCCCAGUCUUCA	179, 200	200	162	4, 0
**38**	AGACUCACAUCCUAUACUCCU	107, 111	111	477	4, 0
**39**	CGCAGCGAAGUAGAUAGACCG	64, 225	225	32	4, 0
**40**	GCCUGUACUCUGGCAACAGUC	42, 154	154	505	4, 0
**41**	CGUAGGUGACGAUUAGCAUAG	106, 51	51	981	4, 0
**42**	CGGCUUGAUACACAGCGUUCG	132, 212	212	117	4, 0
**43**	GUAAUAACUUUCUGCUAUAUA	229, 188	188	626	4, 0
**44**	AUGUAUUGAUGGAGGCUUUCU	71, 83	83	731	4, 0
**45**	AUGAGCCUGCUGUCCAUAUAC	15, 180	180	97	4, 0
**46**	CAAAUGCGGCAUUCUACCAUC	9, 162	162	202	4, 0
**47**	AGGGGCCAUGGACUAUGGAAG	70, 222	222	376	0, 0
**48**	GAACUACUGCUAACUCGAUUC	174, 38	38	96	4, 0
**49**	ACCUCUAGUUGCUCGUGUAGC	105, 224	224	720	4, 0
**50**	CCACAAACAUUUGCAUGCGUU	11, 78	78	1050	4, 0
**51**	GGUGUCAUAUUUCAGUGGUCA	77, 57	57	618	1, 0
**52**	UCCGCCUAAUGUACCACUUUC	144, 34	34	635	4, 0
**53**	AAUGCCAGAUACCUGCUGGUA	203, 208, 209	209	418	4, 3, 0
**54**	ACUAGGUGGAGAGGACUGUCA	114, 227	227	3457	4, 0
**55**	GAUCGUAUGAGGUUCCAUUCC	182, 29	29	70	4, 0
**56**	GUAUUUCUAACAGUUGCCCUU	223, 56	56	927	3, 0
**57**	GUUCCAUGGGCCGCCAUUAGA	87, 122	122	101	4, 0
**58**	UGUCUACAUGGAUAACACUGC	145, 3	3	572	1, 0
**59**	AUGGAAGGCUGGCAUUGAGCU	73, 95	95	551	4, 0
**60**	CCAUCUUGGGCCGGCGCUGCC	76, 227	227	69	4, 0
**61**	GUACUACUUACACGAUUAUAU	97, 209	209	882	4, 0
**62**	CACAGCUUGGUGUGGCAUCAU	142, 227	227	2428	4, 0
**63**	CCGCGUACUUUGCGUUGUUAC	213, 220	220	16	1, 0
**64**	UUUUACUGAAGUCCGAACCUG	218, 48	48	842	4, 0
**65**	AGACCGUGGAGCGAGUGUCCA	113, 225	225	3	4, 0
**66**	UAUUGCGGUAAAGAGCGUCCA	86, 189	189	330	4, 0
**67**	UGAGCCGCAUACUCGCGAUCU	201, 211	211	649	4, 0
**68**	UCAGACUGGGAUCCUGUGAUA	71, 110	110	126	4, 0
**69**	UCCGGGUACCGGUGGUCUCCG	13, 21	21	410	3, 0
**70**	GGUUCAAUAUUGGUCACACUG	174, 103	103	552	4, 0
**71**	CUAAGGUCGAAUUCCACCCAC	217, 215	215	1127	4, 0
**72**	CUCUUAAUAAAGCACUGGAUA	96, 151	151	164	4, 0

**73**	ACCCCGAUAGCUGAAGGGAUG	175, 192	192	512	4, 0
**74**	AGAAACGGACGGAGUGGGCCG	24, 202	202	314	4, 0
**75**	CAUUGGAUAAAACACAGGUGC	197, 200	200	380	4, 0
**76**	UAUCCGGGUUGGGACAAAAGU	88	88	278	0
**77**	CCGCCUCGGGGGAUGUAGGAA	181	181	328	0
**78**	ACAGUCAAGCCAUUAGGGUUG	16	16	304	0
**79**	GGAGUCAUGCAGCUGACCGGU	82	82	662	0
**80**	UAUUCCAUGCUAUGUCCACGG	186	186	354	0
**81**	CCUAACUCAGUCUCCCAUCGC	171	171	59	0
**82**	AUAGUGUUUGUUACUGUAUCC	46	46	657	0
**83**	AAUGCACGGGUUGUUUCGUAU	8	8	364	0
**84**	CCAUACCCGCCAGUGUGUAAU	20	20	164	0
**85**	GCUAAUACUGACUGAGAUCAC	136	136	407	0
**86**	CAAUACAUCCUCUCAUACUUC	66	66	591	0
**87**	GUCCGUCCGCCCUACCAGCAC	4	4	74	0
**88**	GAAUUGCAGCUGUGCAGGGCU	139	139	336	0
**89**	AACAGUGUUGCAGUAAGCACA	52	52	7	0
**90**	ACAGAGUUUGAAUCGAGCCGA	176	176	5	0
**91**	GAAAGUUAGCCCACAUUGGCA	124	124	411	0
**92**	UAGCGGGAGGAACGCAUCCCC	44	44	20	0
**93**	GAUCCAGGUACGGAUGGACAU	195	195	448	0
**94**	UCGCGCCAUUGAGGGCCGUAG	128	128	373	0
**95**	ACACCGUGUGCGUACUCACAG	164	164	407	0
**96**	AAUACAUCUAUGGAUUGGGCA	147	147	271	0
**97**	GUGCUCGCCUCGAUGUUAACC	191	191	558	0
**98**	UGGGACCAUGACCAAUCAGAG	193	193	181	0
**99**	AUUGUUUUAGGCCACAAGCUA	129	129	700	0
**100**	UGUCUAGCAGAGUUCUCUUCC	137	137	507	0
**101**	UCACCAUAGUGCAAAACGGCU	26	26	127	0
**102**	UUUGAGCGUCUCUACAGUCGA	205	205	559	0
**103**	GUGAGCUUUUUAUGCACUCAG	59	59	291	0
**104**	UUUAAUGGGAUAACUUUACAC	40	40	444	0
**105**	UGUCCCACCUACUGUAACUCC	89	89	151	0
**106**	UUCCACGUUUUAUGUUUGGGC	69	69	241	0
**107**	ACUCUUGGUACAAAGAAGGAA	115	115	82	0
**108**	UUGGCUUGUCCUGGUAAUAGC	65	65	1053	0
**109**	GCUUCGGGAUAACGAUGAGUG	183	183	621	0
**110**	CCGAGCCCGCCCUCCUCCGUA	199	199	362	0
**111**	CAUACGUGGCAACUAACACAG	157	157	628	0
**112**	UAAGCAUGGUUCUAGUCAUAA	126	126	70	0
**113**	AUUAGCAAACUCUUCUGAUGC	130	130	658	0

**114**	GCGUCCGCUCGACACGAGCUG	101	101	219	0
**115**	ACUCAUUAAAAGAGUGUUCUC	31	31	1001	0
**116**	AGAACUAUUCCCCUGUUCAAC	190	190	198	0
**117**	UCACUCUUAUGUUGCAACCUG	159	159	700	0
**118**	AAGAACUGGUGGACCCCUUUC	50	50	585	0
**119**	ACUUAGCAGUCAACUGAACGA	39	39	207	0
**120**	UAUAGUAAGCUCGUGGAUAUC	187	187	713	0
**121**	AUAGUUUUAUAUCCGCCGUCG	118	118	419	0
**122**	UCUACCUCUGAUCAGGGAUAU	194	194	538	0
**123**	UUCCGUUCCUGAUUCCUAUAC	2	2	153	0
**124**	UCUCGCUCGAAGCUGAGAACU	54	54	301	0
**125**	AUUUCUUAGCAAUCGGUACCG	53	53	210	0
**126**	GAGGCAUUUAAAGGCACCCCC	177	177	715	0
**127**	UCACGAGAUUGGUUUAAAGUA	99	99	934	0
**128**	GAACUUACAGUGCGUCGUCGG	23	23	454	0
**129**	UAUACGACAGCUAAAAUGACC	104	104	260	0
**130**	UCUGUGGUACCCUCCAGACCG	156	156	383	0
**131**	CUAUUGUAUGGUUUCUGCACC	172	172	274	0
**132**	UGUAAUUCGCUGCAUUAUGUU	196	196	565	0
**133**	UAGCCCGCUUGGAACUUCUGA	131	131	86	0
**134**	CGAACCGAUUAUUCGAGUGUG	117	117	366	0
**135**	CAAAUGACUGGAACCAUACUC	19	19	247	0
**136**	AUUAGAGAACAGUCCCCAUUA	109	109	261	0
**137**	UAAUUAUCUCGAAAUAGGGCG	116	116	690	0
**138**	CGUUAACGAUCGGGGUAGGAC	149	149	207	0
**139**	ACAUGCUGCUCUGGUUCGCGU	163	163	61	0
**140**	UAUGCUCCAGUCAUGCAGGAC	150	150	311	0
**141**	ACAUUCAGUUUGUUGAGUCGU	185	185	159	0
**142**	GAUUGCUUUUGCGCAUUCUCG	198	198	145	0
**143**	AGUGCAUCAUGGAGACUGCGC	93	93	405	0
**144**	AAACACGCCACGUGGCAAGGA	161	161	211	0
**145**	CUUGUGCAACCUCCGUUUAGA	141	141	281	0
**146**	UAUUCGGGCGCUCUCAGAGAA	108	108	206	0
**147**	CAUUUAGGAUAGUAAUUAACC	214	214	658	0
**148**	UUAAAUGAGUCAUCUUGUAAC	41	41	1000	0
**149**	ACGAUCUAACUUGACACCGGU	140	140	516	0
**150**	CGUGGGCCUGGUGCAUUAAAC	47	47	469	0
**151**	CCUCACCGAGCAUCCUUAAGG	98	98	867	0
**152**	CUGAACGCGAGACCGCUCGUC	135	135	28	0
**153**	ACAUGCCCUAAUUUCCUACGA	1	1	799	0
**154**	UAUCUGGGUAUUAGAACCCCU	228	228	555	0

**155**	UACGAUCACACAGCGUAAAGA	169	169	263	0
**156**	AGAGCCCUAGGGUGCUUAUCA	100	100	99	0
**157**	UCUCUUUUUAGAGAGACCACG	17	17	286	0
**158**	UUUAUCCUUAACUAAGCGGCC	72	72	790	0
**159**	AAAGCAAUUCUGCUUGGACUG	138	138	443	0
**160**	CCUGGCAUUAUGUGAAAGGAU	152	152	608	0
**161**	AAUAUUUGACUUCGAACUAAC	18	18	263	0
**162**	CGAGACUGAAGGCCGUGGCAA	22	22	125	0

*** The Transcription factor numbers and postions of the qualified sequences which the selected qualified siRNAs are derived from.**

**** The mismatch numbers between qualified siRNA and its target genes.**

Table S3

Parameters: d_T=3, d_N=5, L=21

Number of non-redundant qualified siRNAs: **487**

Number of qualified siRNAs selected in the first round of RNAi analysis: **142**

Number of candidate-genes examined in the first round of RNAi analysis: **229**

Average examination frequency of each gene: **1.15283842795**

No.	Selected qualified siRNA	Target genes of qualified siRNA (TF number)	*Qualified sequence		**Mismatch numbers
			TF number	Position	
1	UGAUCUCUAUCUUCCCCCUCC	211, 210, 207, 209, 208, 75, 206	206	149	3, 2, 1, 1, 2, 3, 0
2	UAUCGUAAGCUCGGGCCGCGG	167, 112, 74, 175	175	167	2, 3, 3, 0
3	CAUAAGCCUUCUUCAGGAGGC	68, 58, 104, 206	206	224	3, 3, 2, 0
4	ACGUUUAAGAAGCUUCUCCGC	103, 77, 57	57	114	3, 2, 0
5	AGAAACUCAACUUGCCGCUGC	159, 67, 146	146	188	2, 3, 0
6	CUGGCGACUUGAGUUGGUGUC	120, 49, 5	5	117	1, 3, 0
7	UUUAGAACAUGCGUGGGAAUG	144, 3, 145	145	310	3, 1, 0
8	UUCUGCCCAUAUUUCCUCCAC	187, 106, 157	157	503	2, 3, 0
9	UAUUGUUCUUUAUAAAGACCU	46, 70, 222	222	576	3, 0, 0
10	UCUUUCUUGAUGACCUUGAUA	122, 6, 158	158	871	3, 0, 0
11	CUUCCUGCAUGCUUAACUAUC	86, 45, 191	191	62	3, 3, 0
12	UUCAGCCACCGAAGCCGGCAG	220, 69, 221	221	267	3, 3, 0
13	UAUGAACCAGUUAGAGACCUG	204, 143, 32	32	197	2, 3, 0
14	GGUAUCGAAGGUGCCAAGCCA	149, 102, 112	112	588	3, 3, 0
15	UAUUUGAGCCUUAUAAAUUUA	116, 36, 220	220	983	3, 3, 0
16	UGUUUUCCGGCAAGUUUGUUG	133, 168	168	218	1, 0
17	UCUCACUCUUCACUUUCCUUC	65, 73	73	582	2, 0
18	ACAAUAAUGAAAUGCAAUGGG	127, 170	170	496	0, 0
19	GAUGCUCGUGCCACCUCGGAG	94, 151	151	685	2, 0
20	CAUAGCAUAUUGUUUUCUAAA	12, 223	223	914	3, 0
21	CCAAUCAAUCAACCAAAUACG	166, 139	139	677	3, 0
22	CGCGGCAGCGGGUGCCUCCGU	78, 30	30	409	3, 0
23	UCAUCGAGUAUGAAAAGAACG	34, 212	212	1216	3, 0
24	AGUCAAUACCCGAACCAACAC	163, 161	161	282	1, 0
25	CCUCUCGGCUUCCACAUGGUU	205, 190	190	2	2, 0
26	CCAUUAGAUGGAGACGACUGU	227, 114	114	170	3, 0
27	GCUCCGCAAAGAACGGCCAAG	215, 216	216	1294	3, 0
28	CCUAGACAACCAGUGAAACUC	177, 178	178	131	3, 0
29	ACUUACAAGUCCCAGUCUUCA	179, 200	200	162	4, 0
30	CUUCUUAGAGCGAGAGAGAGG	110, 213	213	80	3, 0

31	CAAAGUUCUACUAAAUUAAGU	71, 131	131	242	3, 0
32	CAAAUCAACAUUCAAGGUAAU	183, 154	154	690	3, 0
33	CGUGAUGCUCCGGGUACCGGU	15	15	228	0
34	GAAGUAUAGAUUAUAAGAGAA	134, 61	61	618	3, 0
35	UCGUAGCGGGAGAGAGAAGAG	193, 54	54	136	3, 0
36	CUCGCAUCUCCUCCUUUCCGC	124, 1	1	552	3, 0
37	AGUUCGGUUCUCCGCCGACGC	218, 24	24	502	3, 0
38	GCAAUCGUUCUGAAGACAGGC	150, 229	229	369	3, 0
39	GCUUCUAUCUCCUCAUCAUAC	66, 56	56	636	3, 0
40	AACCACUUAUCGAUGCAAUCU	11, 14	14	68	3, 0
41	GUAUCUGGUGAUAUCAAGAGA	111, 174	174	13	2, 0
42	UCCUCCUCCAUACCUUUCCUU	214, 186	186	157	3, 0
43	AAUUGAUGGUGGCAUAAAUCC	15, 29	29	586	3, 0
44	GAAACAGAGAAGGUGAGCUUU	4, 96	96	691	3, 0
45	UGAGGCCUUUGCCGCUUGGUU	22, 197	197	258	3, 0
46	CCUCCAGACCGGCAUUUUUGA	109, 156	156	373	3, 0
47	CUUGGAGUAUCAGCAUGUUGC	135, 201	201	524	2, 0
48	CUUCAGCUGCGGCCAUGGCUG	152, 162	162	750	3, 0
49	GAACCCAAAUGACAUUCAUGA	10, 172	172	463	3, 0
50	UCGUUCACAAACAUUGCAGCU	43, 63	63	175	3, 0
51	CCCAGCACACUCCCUCCUCCC	9, 164	164	830	4, 0
52	CAACAUCAGCUCCUCACCGAA	98, 95	95	791	3, 0
53	GCUUCAGCUUCGUUCUCGCUC	125, 37	37	618	3, 0
54	CAUUUAUCCUUCAGGUCAACA	19, 184	184	305	3, 0
55	UUUGGAAAGGUCUUCCAACGU	39, 28	28	576	3, 0
56	CGCAAUAACCAACAAAUAAUA	23, 85	85	937	3, 0
57	CCCUUAAUUCUUCUUCAGAAG	129, 64	64	355	3, 0
58	AAGAUGAUUAAAGAAGCUAGG	130, 89	89	604	3, 0
59	UCUGUUCCUCGAGCCAUCGAG	142, 7	7	22	2, 0
60	GUUGAAAAUAGCGAGGAACUU	79, 83	83	1289	3, 0
61	GAGUACUGCUUCUUCGUCAAC	87, 91	91	874	3, 0
62	AGAACAACGUAAGGAUCGCUG	48, 188	188	122	3, 0
63	AAGAAAAGAAGCAUCCCAUAA	138, 97	97	441	3, 0
64	AGAGCUCGAUGAGCUCGAGCU	50, 20	20	306	3, 0
65	AAAGCCUUGUCUUUUUUCUGC	76, 196	196	255	3, 0
66	UCUCAGUGAUCCAGCCAUCAA	224, 100	100	445	3, 0
67	UCUGUGGUUACAGAAGAAUGC	107, 123	123	917	3, 0
68	CAGUAGGAUGGAAUCUGAAAC	155, 33	33	195	3, 0
69	GAUUUCCUGCUUCUAGACAUC	26, 141	141	310	3, 0
70	AGCUCCUUGGCCUUCUUAAGA	207, 68, 60	60	170	2, 3, 0
71	UCACAAUGUCUUCUGGUUUUU	86, 45, 189	189	507	3, 3, 0

**72**	AGGUGCCAAGCCAAGCCCGGU	112, 90	90	239	3, 0
**73**	AUUUUUGGACCAUGUCAUCAA	103, 93	93	63	0, 0
**74**	CAUCGUCAUGAUGGGCUUUUC	53, 73	73	431	3, 0
**75**	UUACGCCAGUGGCGCUACCGC	102, 160	160	59	3, 0
**76**	GGUUCAUGGACAAAAAGCUCC	116, 165	165	904	2, 0
**77**	AGCUGGGAGGGGUAGGAGGGG	99, 227	227	3437	3, 0
**78**	CCGGCUUUUCUUGGGAGAAGG	219, 106	106	95	3, 0
**79**	AAAAUUCAUCACAUUUAGAUA	136, 212	212	1689	3, 0
**80**	UAGCUUUUUCGGGGCCUCCGA	68, 105	105	31	3, 0
**81**	UGGGCUUUCUGUGCUGGGGGG	228, 204	204	727	3, 0
**82**	GCAAUGUACAUAAAUAUGAGA	47, 71	71	1796	3, 0
**83**	GUGUGAGCGAAGAAGCACACA	55, 164	164	500	3, 0
**84**	UCACGAAGGCCGCCGUCGAUG	212, 217	217	1055	3, 0
**85**	UGAUGAAAAGUUCGGUUCUCC	31, 24	24	510	3, 0
**86**	GCAAGCAAAGCAAAAUAUAUC	177, 81	81	829	3, 0
**87**	CCAUCUCAACUAGUAUGAAAA	212, 171	171	34	3, 0
**88**	AAUGCGAGCACUUCCUCGUCA	62, 120	120	166	3, 0
**89**	GGUCAGCUGGAUUUGAAGGAA	192, 150	150	657	3, 0
**90**	UCUCUGAAUUCAACUUGAUCU	72, 98	98	258	3, 0
**91**	CAUCAGCAGAAGUGGUGAUAA	98, 41	41	446	3, 0
**92**	CCUUGAUCGGCUUCUGACCAU	106, 51	51	870	1, 0
**93**	CGGCAAAAACUGAAAUCUUUU	132, 95	95	824	3, 0
**94**	UUAUAACGAUAAUACAGACGC	40, 20	20	738	3, 0
**95**	CUUGAACUCUGAAUAACUUCG	92, 223	223	1118	3, 0
**96**	AUUAAGAAACCUAUCCUCUAU	121, 130	130	1181	3, 0
**97**	UAACACUUUAUAGCAUUACAU	118, 89	89	924	3, 0
**98**	GUGUAAUCCCCCCGUCACUCC	173, 20	20	207	3, 0
**99**	GGGGGAAGUUGCGGUAGAGCU	199, 227	227	1596	3, 0
**100**	UGUGGACCUACAGAGGGGGCA	44	44	329	0
**101**	AAUAAAUGGCUCAUUCUUCAA	203	203	721	0
**102**	CUUAAAAAUUGAUCAUUUCAU	108	108	661	0
**103**	AGAGUCUUAACAAAGAUUUGC	226	226	280	0
**104**	AUAAACACUCUCUACCUCUGA	194	194	548	0
**105**	AGACGAGGUAGAAUUUUUUGA	80	80	299	0
**106**	UCAUAAAAACCAACUCUCUCA	82	82	614	0
**107**	GAUUUGUCUAUGCUAAUAACA	119	119	295	0
**108**	GAUCCGAUCUGAAUCCCAAGA	148	148	455	0
**109**	UGUGCAGAAAACUGGUCCAAU	42	42	359	0
**110**	GUUGAUCCUGGAAGGCACAUU	16	16	83	0
**111**	GAAACUUGAGAAUCACCUUCC	198	198	362	0
**112**	GAAUAUGAGUGAUUCUACUCC	128	128	734	0

**113**	CCUAUAAGGAAUGGGGAAAAC	182	182	74	0
**114**	AGAUUCGUGGCCUUUGCAAAA	137	137	793	0
**115**	AUAAAUACUGACAGCAGCUGA	84	84	123	0
**116**	AAAUCUUUAAAAUAGCAGAAC	8	8	601	0
**117**	CUAGUUGUACACAUAAUAUAC	35	35	575	0
**118**	GAACUUCCUCCCUUAAUGCCU	180	180	137	0
**119**	CCUGCUGCGCCUGGAUACUGC	2	2	237	0
**120**	CGAGAGGCUUACUCGUUGGUA	169	169	420	0
**121**	CAUUAAUAAAAGAAAGUCCAC	59	59	462	0
**122**	GUAACAUCUAAAAUCACAUAC	17	17	369	0
**123**	UCACGGCUGGCGAUUGCAGAA	202	202	369	0
**124**	AAAAAAACUAAAUGUAACUGC	147	147	667	0
**125**	CCAAUAUUUGACUUCGAACUA	18	18	265	0
**126**	UAUUGAAGAAUCAUAUUCAGC	101	101	721	0
**127**	ACUUCUCGUAACAUAUGUUGG	38	38	7	0
**128**	UAGUUCCCUCAUGGAUCAUCC	126	126	35	0
**129**	AGAGUAAUGAGCUUGAAGCAA	52	52	127	0
**130**	GCUGGGAUUCACAAGGCUUUC	181	181	638	0
**131**	UGAGGUUUUUCAAGUCAUACU	115	115	58	0
**132**	UGAUUCGGGUUGGAACUUCAU	88	88	298	0
**133**	ACGGCAGAGAGGACAAGUGUC	176	176	96	0
**134**	GAUGAAAGAAGAUCCUUCAAU	117	117	8	0
**135**	CCAAGCUUAAAUAACAAAGAA	225	225	1307	0
**136**	CUAACCCUUAACAUCAUACGA	153	153	724	0
**137**	CGACGAACCGUACUCCAGAUC	195	195	465	0
**138**	GUAAAUGUUGAGCACACUGGU	25	25	833	0
**139**	AAAUAUAUGAAAGAAUCACUA	27	27	617	0
**140**	CCAGUCGAGGCGAGAUGCAGG	185	185	40	0
**141**	AUUUAGGCUUUAAAUACACAA	113	113	272	0
**142**	CCGGCGUUUGCCACUUAGGGC	140	140	450	0

*** The Transcription factor numbers and postions of the qualified sequences which the selected qualified siRNAs are derived from.**

**** The mismatch numbers between qualified siRNA and its target genes.**
